# Synchrotron imaging of dentition provides insights into the biology of *Hesperornis* and *Ichthyornis*, the “last” toothed birds

**DOI:** 10.1186/s12862-016-0753-6

**Published:** 2016-09-23

**Authors:** Maïtena Dumont, Paul Tafforeau, Thomas Bertin, Bhart-Anjan Bhullar, Daniel Field, Anne Schulp, Brandon Strilisky, Béatrice Thivichon-Prince, Laurent Viriot, Antoine Louchart

**Affiliations:** 1 Centre National de la Recherche Scientifique, Unité Mixte de Recherche 5242, Institut de Génomique Fonctionnelle de Lyon, Equipe évo-dévo de la denture chez les vertébrés, Ecole Normale Supérieure de Lyon, Université Lyon 1, 46 Allée d’Italie, 69364 Lyon cedex 7, France; 2 UMR CNRS/MNHN 7179, “Mécanismes adaptatifs: des organismes aux communautés”, 57 rue Cuvier CP55, 75005 Paris, France; 3 ESRF—The European Synchrotron, 71, avenue des Martyrs, CS 40220, F-38043 Grenoble Cédex 9, France; 4 Department of Geology and Geophysics and Peabody Museum of Natural History, Yale University, New Haven, 06520 CT USA; 5 Natuurhistorisch Museum Maastricht, De Bosquetplein 6-7, NL-6211 KJ Maastricht, The Netherlands; 6 Present Address: Naturalis Biodiversity Center, Darwinweg 2, 2333CR Leiden, The Netherlands; 7 Royal Tyrrell Museum of Palaeontology, P.O. Box 7500, Drumheller, T0J 0Y0 AB Canada; 8 Centre National de la Recherche Scientifique, Unité Mixte de Recherche 5553, LECA, Equipe Paléo-Génomique, and Palgene (CNRS/ENS de Lyon), Ecole Normale Supérieure de Lyon, Université de Lyon, 46 Allée d’Italie, 69364 Lyon cedex 7, France

**Keywords:** Dental attachment, Dental replacement, Dinosaurs, Fossil birds, Growth, Histology, Microstructure, Paleobiology, Teeth

## Abstract

**Background:**

The dentitions of extinct organisms can provide pivotal information regarding their phylogenetic position, as well as paleobiology, diet, development, and growth. Extant birds are edentulous (toothless), but their closest relatives among stem birds, the Cretaceous Hesperornithiformes and Ichthyornithiformes, retained teeth. Despite their significant phylogenetic position immediately outside the avian crown group, the dentitions of these taxa have never been studied in detail. To obtain new insight into the biology of these ‘last’ toothed birds, we use cutting-edge visualisation techniques to describe their dentitions at unprecedented levels of detail, in particular propagation phase contrast x-ray synchrotron microtomography at high-resolution.

**Results:**

Among other characteristics of tooth shape, growth, attachment, implantation, replacement, and dental tissue microstructures, revealed by these analyses, we find that tooth morphology and ornamentation differ greatly between the Hesperornithiformes and Ichthyornithiformes. We also highlight the first Old World, and youngest record of the major Mesozoic clade Ichthyornithiformes. Both taxa exhibit extremely thin and simple enamel. The extension rate of *Hesperornis* tooth dentine appears relatively high compared to non-avian dinosaurs. Root attachment is found for the first time to be fully thecodont via gomphosis in both taxa, but in *Hesperornis* secondary evolution led to teeth implantation in a groove, at least locally without a periodontal ligament. Dental replacement is shown to be lingual via a resorption pit in the root, in both taxa.

**Conclusions:**

Our results allow comparison with other archosaurs and also mammals, with implications regarding dental character evolution across amniotes. Some dental features of the ‘last’ toothed birds can be interpreted as functional adaptations related to diet and mode of predation, while others appear to be products of their peculiar phylogenetic heritage. The autapomorphic *Hesperornis* groove might have favoured firmer root attachment. These observations highlight complexity in the evolutionary history of tooth reduction in the avian lineage and also clarify alleged avian dental characteristics in the frame of a long-standing debate on bird origins. Finally, new hypotheses emerge that will possibly be tested by further analyses of avian teeth, for instance regarding dental replacement rates, or simplification and thinning of enamel throughout the course of early avian evolution.

**Electronic supplementary material:**

The online version of this article (doi:10.1186/s12862-016-0753-6) contains supplementary material, which is available to authorized users.

## Background

Modern birds represent an extraordinarily diverse clade, and exhibit tremendous disparity in their feeding ecologies and diets [1, 2]. Remarkably, this dietary disparity is realized despite the fact that crown birds lack teeth. Although genomic evidence suggests a single evolutionary loss of teeth prior to the extant avian radiation, and as early as c. 116 Ma ago [3], their Mesozoic relatives generally retained teeth [4]. In particular, the closest stem group relatives of crown birds, representatives of the extinct clades Ichthyornithiformes and Hesperornithiformes, retained teeth in the major parts of the jaws [4–6]; however, which of these clades represents the immediate sister to crown birds remains contentious (e.g., [7, 8]). Together with crown birds, the Ichthyornithiformes and the Hesperornithiformes form the Ornithurae (ornithurine birds). Representatives of these crownward stem ornithurines are known from the Late Cretaceous (c. 100 to 66 Ma) of northern and central America —for the Ichthyornithiformes— and the Holarctic region —for the Hesperornithiformes (e.g., [4, 6, 8–11]). None of the toothed bird lineages survived the 66 Ma extinction crisis [4].

Here, we present the first detailed investigation into the dentition of both the Ichthyornithiformes and Hesperornithiformes. We focus our analyses on *Ichthyornis dispar* and *Hesperornis regalis* (by far the best documented species respectively in the Ichthyornithiformes and the Hesperornithiformes), as well as other specimens of bird teeth or non-avian theropod teeth. In order to characterize the dentition of these fossils we employ a variety of visualization techniques, including both synchrotron and conventional x-ray microtomography, and other non-destructive and non-invasive technologies such as scanning electron microscopy.

Vertebrate dentitions exhibit numerous quantifiable characteristics, from the scale of the jaws down to that of the crystallites constituting mineralized dental tissues such as enamel and dentine. The excellent preservation potential of mineralized dental tissues (including enamel, dentine, cementum, periodontal tissues, alveolar bone, etc.) facilitates myriad comparisons among extant and fossilized vertebrates. Among other implications of interest to evolutionary biologists, such comparisons may shed light on the evolution of tooth distribution patterns, tooth implantation modes, and tooth replacement during life (e.g., [12–16]), their microstructure and histology [17–19], and their growth dynamics [20–22]. Based on such insights, fossilized dental remains may provide a wide range of information regarding an extinct organism’s paleobiology, diet, mode of food processing, mode of growth, metabolism, and phylogenetic position.

Characteristics of tooth macroevolution have been studied in detail across tetrapods, providing a rich context for comparison with birds. For instance, among and beyond the archosaurian relatives of birds, macroevolutionary patterns have been studied in detail in crocodilians, non-avian dinosaurs, and early amniotes. These studies cover many aspects of dental biology across these taxa, including tooth morphology [23–27], the microstructure of dental tissues (particularly enamel) [17, 28–30], tooth implantation, development and replacement [31–35], and the growth rhythms of dentine [20, 21]. However, bird dentitions remain comparatively understudied [36, 37]. Although several authors (e.g., [13]) have treated Mesozoic avian dentitions, nearly all investigations have thus far remained macroscopic, generally with little illustrative support, and frequently contradictory interpretations. Numerous aspects of avian dental biology remain poorly characterized, including tooth morphology, microstructure and histology, tooth attachment and implantation mode, degree of thecodonty, presence or absence of alveolar bone, mode of tooth replacement, and growth rhythms.

Our observations on avian teeth are principally compared with dental characteristics of non-avian archosaurs and mammals. Our results allow us to both characterize the dentition and teeth of *Hesperornis* and *Ichthyornis*, and to present new identification criteria, useful for the diagnosis of isolated fossil teeth. Moreover, based on these data, we propose new interpretations of the paleobiology, growth, and metabolism of *Hesperornis* and *Ichthyornis*, which together bring us closer to a more detailed understanding of how modern birds came to be.

## Results

### Dental morphology

#### Hesperornis

**Fig. 1:**
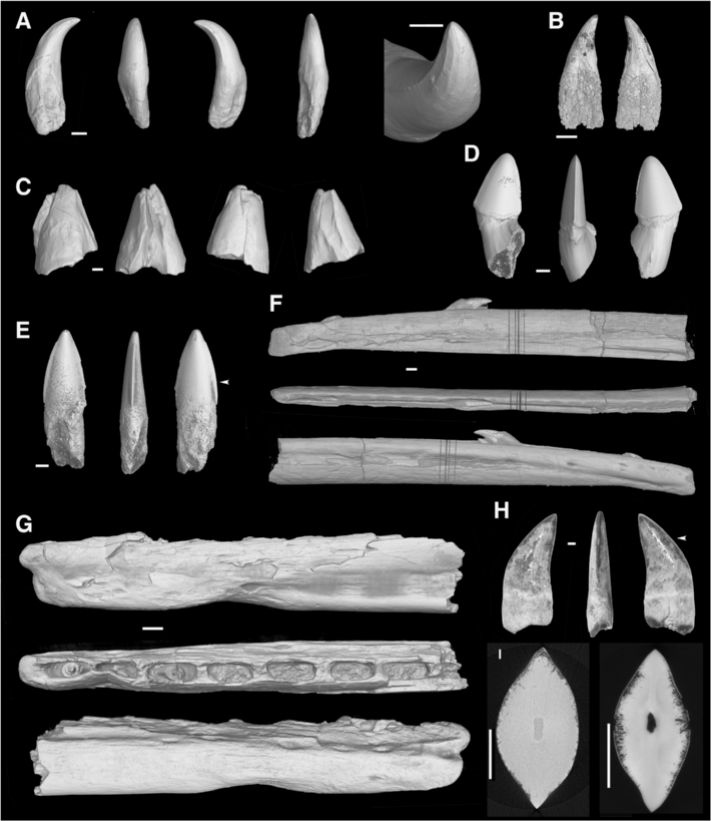
X-ray microtomographic views of the studied teeth or dentary fragments with teeth of *Hesperornis regalis*, *Ichthyornis dispar*, and Ichthyornithiformes indet. **a** Isolated tooth of *Hesperornis regalis* YPM.1206B, complete tooth. From left to right: lingual, mesial, labial and distal views, and apical part showing fluted ornamentation. **b** Virtually extracted tooth TH_2_ from *Hesperornis regalis* dentary YPM.1206A, lingual (left) and labial (right) views. Isolated teeth of *Ichthyornis dispar*: **c** YPM.1460, damaged tooth tip fragment, four views; **d** UAM_PV93.2.133_1, nearly complete tooth, from left to right: lingual, mesial and labial views; **e** UAM_PV93.2.133_2, nearly complete tooth, from left to right: lingual, mesial and labial views (small arrow shows level of horizontal section in **i**). Incomplete dentaries of: **f** *Hesperornis regalis* YPM.1206A, right dentary mesial portion, from top to bottom: lingual, occlusal and labial views; **g** *Ichthyornis dispar* YPM.1775, right dentary portion, from top to bottom: lingual, occlusal and labial views. **h** Isolated tooth of Ichthyornithiformes, NHMM/RD271, from left to right: labial, distal and lingual views; small arrow shows level of horizontal section in **i. i** comparison of crown horizontal section shapes of *Ichthyornis dispar* UAM_PV93.2.133_2 (left) and Ichthyornithiformes indet. NHMM/RD271 (right). All x-ray microtomography external views (synchrotron microtomography except **d**, **e** and (I,left) (conventional microtomography), and (H) (digital photography). Scale bars, **a**, **b**, **c**, **d**, **e**, **h** and **i** 0.5 mm, **f** 2.5 mm, **g** 0.75 mm

**Fig. 2:**
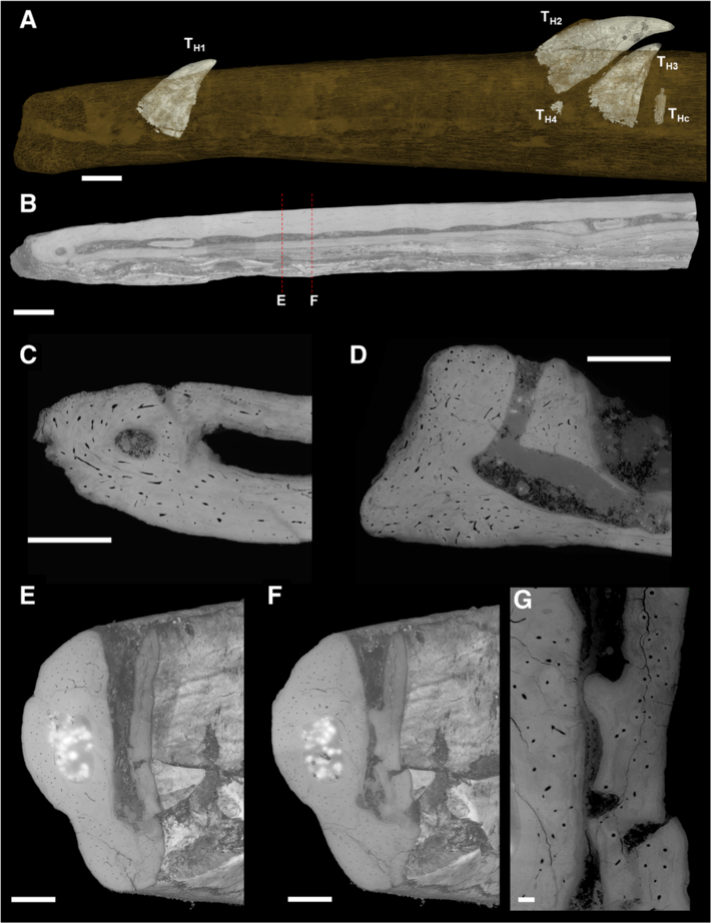
Synchrotron x-ray microtomographic images of *Hesperornis* dentary. Anterior part of the dentary of *Hesperornis regalis* YPM.1206.A, showing the implantation location of the preserved teeth. **a** Lingual view showing by transparency four teeth (T_H1_ to T_H4_) and some preserved cementum of one tooth (T_Hc_) present in the groove. **b** Horizontal volume section of the dentary showing the groove and its constriction, in occlusal view. **c** Horizontal section at mesial end. **d** parasagittal section along groove at mesial end. A small hole, that we interpret as a minute alveolus, is visible at the dentary mesial extremity (**c**, **d**). It communicates with the groove through a ventrally situated “tunnel”. **e** Transverse volume section through the middle of the insertion location of a (missing) tooth. **f** Transverse volume section through a constriction of the groove adjacent to (and delimiting) the preceding. Labial to the groove is the medullary cavity running parallel along the jaw. **g** Virtual transverse section of the same constriction as in **f**: it appears that it is shaped as a bulge of jaw bone tissue. Scale bars **a**, **b** 2.5 mm, **c**, **d**, **e**, **f** 1 mm, **g** 0.15 mm

The observed teeth of *Hesperornis* are unicuspid, with a pointed, acute crown tip, and show different degrees of crown tip distad recurvature (i.e., caudad) resulting in a somewhat hooked shape. The isolated tooth YPM.1206B is very recurved (Fig. 1a). The teeth preserved in the lower jaw (dentary YPM.1206A; Fig. 1b) are less recurved than YPM.1206B (and more compressed labio-lingually, due to postmortem distortion). The YPM.1206A dentary teeth appear to have been *in situ* at death, having been subsequently displaced slightly within the dentary (see implantation), resulting in their present orientation in which they are strongly slanted distally (i.e., toward the caudal or posterior direction). Their inclination is more pronounced in the mesial (i.e., rostral or anterior) part of the dentition (Fig. 1f), which might be a biological feature despite postmortem displacement being likely for the tooth T_H2_, which has come to overlie the tooth T_H3_ (Fig. 2a). The teeth exhibit thin but marked basal-apical ridges on their crown surface (Fig. 1a), and moderately prominent cutting mesial and distal carinae on the crown (Fig. 1a). The latter mesial and distal carinae do not reach either the apex or the neck of the tooth, and they bear no serrations.

The major part of each of the tooth roots within the YPM.1206A dentary is missing, and the crowns largely do not emerge from the jaw tomia (Figs. 1b and 2a). We interpret this as teeth being preserved at a non fully-grown stage (see Discussion-Replacement), where the roots are unfinished and teeth only starting to emerge at the time of death. In contrast, the isolated tooth YPM.1206B has most of its root preserved. This root is greatly expanded, higher than the crown, and there is a mesio-distal expansion at the transition from crown to root, and not a constriction.

#### Ichthyornis

**Fig. 3:**
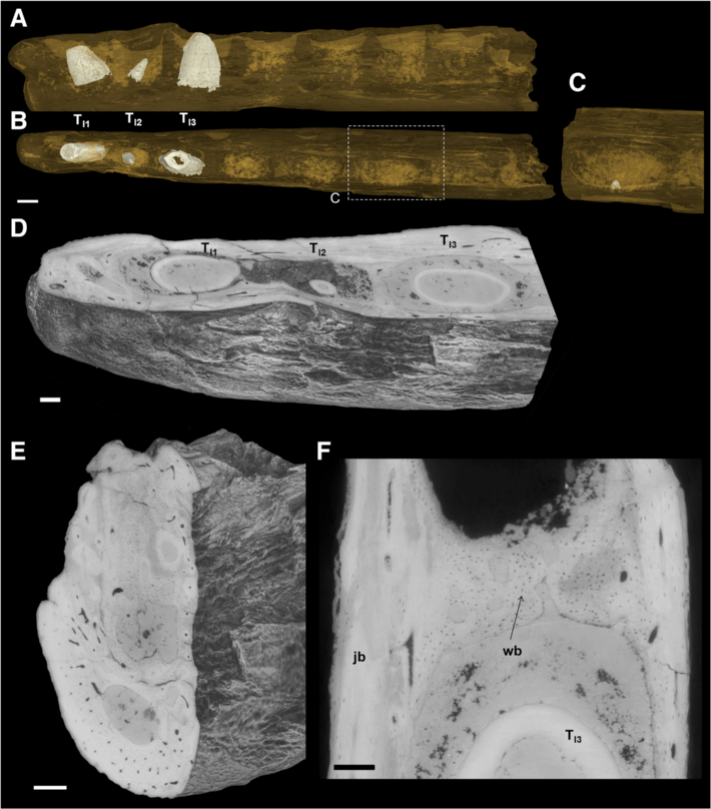
Synchrotron x-ray microtomographic images of *Ichthyornis* dentary. Dentary of *Ichthyornis dispar* YPM.1775, indicating the implantation of the three teeth in the anterior part of the mandible and an additional, small tooth. **a** Lingual view. **b** Occlusal view. **c** Insert at higher magnification showing a small tooth preserved in the distal part. **a**, **b** and **c** are volumes rendered with a level of transparency, and showing segmented teeth in volume (in grey). The tooth germ shown in **c** is not segmented and highlighted in **a** and **b. d** Horizontal volume section of the mesial part of the specimen, exposing the insertion of teeth in sockets. The alveolar bone is clearly seen in transverse volume section **e** and in horizontal section **f** at higher magnification, with woven bone tissue (wb) and lamellar bone forming the septa and the main jaw bone (jb), respectively. Scale bars **a**, **b** 0.75 mm, **d**, **e**, **f** 0.25 mm

The observed *Ichthyornis* teeth are not as recurved as *Hesperornis* teeth. The more complete *Ichthyornis* teeth studied (Fig. 1d, e) have an acute, pointed crown and are essentially straight, not recurved. The tooth UAM_PV93.2.133_1 (Fig. 1d) presents a very general recurved appearance, but does not exhibit the hooked crown of *Hesperornis* teeth: in *Ichthyornis* the tooth recurvature arises from a distal inclination of the crown relative to an upright root, but the crown itself shows straight mesial and distal borders (inclined distally). There is therefore a clear angle between root and crown in UAM_PV93.2.133_1 (Fig. 1d). A similar angle is barely discernible in the first and third teeth present mesially in the dentary YPM.1775, in which most of each root is lacking (Figs. 1g and 3a, b). While some of the observed teeth from the dentary of *Ichthyornis* and the badly preserved tooth apex YPM.1460 (Fig. 1c) are quite conical, several isolated teeth are labiolingually compressed with sharp, blade-like mesial and distal cutting edges (Fig. 1d, e). There are no serrations on these carinae. The surface of the teeth of Ichthyornithiformes is devoid of surface ornamentation, unlike the teeth of *Hesperornis*. Any slight grooves present on the dentary teeth of *Ichthyornis* appear to represent post-mortem cracks due to alteration or diagenesis.

Like in *Hesperornis*, all the teeth *in situ* in the *Ichthyornis* dentary (YPM.1775) lack nearly all of their roots (Figs. 1g and 3a, b); again we interpret this as a consequence of unfinished growth of these replacement teeth at the time of death (roots not grown, and crown not emerging or barely), while the preceding generation of teeth was shed (see Discussion-Replacement).

##### Other, comparative teeth

The ornithurine tooth NHMM/RD271 (Fig. 1h) is most similar to *Ichthyornis* in shape. The distad crown inclination, due to an offset angle between the root and the crown, is also seen in the more mesially-situated *Ichthyornis* teeth (e.g., Fig. 1d). NHMM/RD271 is strongly compressed labio-lingually, and exhibits sharp, unserrated cutting mesial and distal edges (Fig. 1h, i). Its horizontal section is of similar shape to that of an *Ichthyornis* tooth (Fig. 1i). In NHMM/RD271, crown height is 4.90 mm, length at crown base is 2.80 mm, and width at crown base is 1.23 mm.

**Fig. 4:**
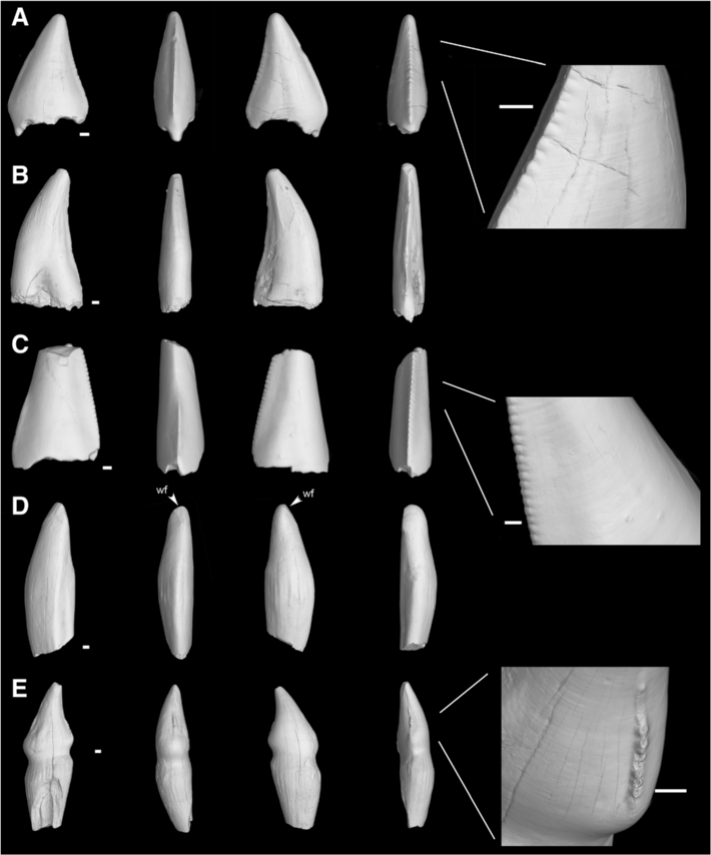
Synchrotron x-ray microtomographic views of the studied teeth of Aves and other, theropod taxa from the Late Cretaceous. **a** TMP 1986.030.0039, tooth crown. **b** TMP 1986.052.0054, tooth crown with small apical part of root preserved. **c** TMP 1994.031.0032, tooth crown with broken tip. **d** TMP 1996.012.0040, tooth with crown and two thirds of root (broken). **e** TMP 1989.103.0025, tooth with crown and most of root preserved (broken). Whole views are, from left to right, in **a** and **c**: labial, mesial, lingual and distal; in **b**, **d** and **e**: lingual, mesial, labial and distal. Magnified views show the diverse shapes and densities of serrations of the distal carina in **a**, **c** and **e**. wf, wear facet. All synchrotron x-ray microtomography external views. Scale bars, 0.25 mm

**Fig. 5:**
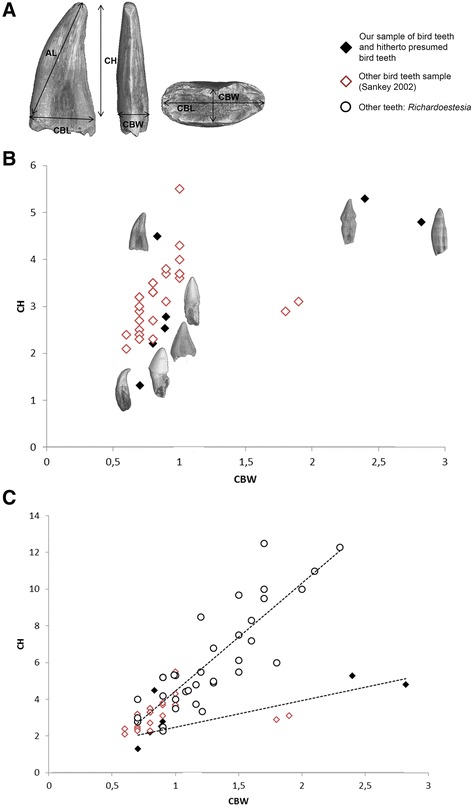
Discriminant morphometric parameters of the studied avian and non-avian teeth. **a** Morphological parameters used to study tooth shape (modified from [38]). AL, apical ‘length’; CBL, crown base length; CH, crown height; CBW, crown base width (see Additional file 1: Table S1 and Additional file 2: Fig. S1). **b** Comparison of the studied teeth with published data on specimens of the non-avian theropod genus *Richardoestesia* [25] reveals that attested and putative Late Cretaceous birds overlap with non-avian specimens in terms of height vs. length or width, with comparable variance. **c** The slight difference between teeth of non-avian taxa otherwise morphologically close to avian ones, such as *Richardoestesia* [38], and teeth positively identified as avian (our *Hesperornis* and *Ichthyornis* specimens) corresponds to the degree of crown base labio-lingual enlargement relative to height, i.e., ratio of crown width at the level of the neck versus height. The tooth TMP 1989.103.0025 is here considered as presumed avian (eventhough this is debated, with some recent publications proposing *R. isosceles*; see Results and Discussion)

Among the comparative teeth from Alberta, the following were hitherto identified as “Aves indeterminate” based on external gross morphology and proportions: TMP 1986.030.0039, TMP 1986.052.0054, TMP 1994.031.0032, and TMP 1996.012.0040 (Fig. 4). We observe fine serrations on the carinae of the crown in TMP 1994.031.0032 and TMP 1986.030.0039 (Fig. 4a, c). TMP 1996.012.0040 is large, with a faint constriction at the crown base, and shows what we interpret as a wear facet at the crown apex (Fig. 4d). TMP 1989.103.0025, recently identified as the coelurosaurian theropod *Richardoestesia isosceles* [27, 38], is as large as TMP 1996.012.0040, shows a marked constriction between crown and root, and displays a limited zone of the carina with minute and peculiar serrations (Fig. 4e). In addition, we compared morphometric parameters of the studied teeth along with published data on other coelurosaurian teeth [25, 27, 38] (Additional file 1: Table S1). Avian teeth and other theropod teeth show important overlap in many dimensions and ratios (Fig. 5; and see Supporting Information files Additional file 2: Fig. S1). Only the graph showing crown base width versus crown height (Fig. 5) displays a discriminant criterion. But the two teeth defining this pull out are TMP 1996.012.040 and TMP 1989.103.0025, there provisionally considered presumed avian teeth, and which are the two largest teeth of the sampling (see Discussion). The avian, ornithurine teeth (including *Ichthyornis* and *Hesperornis*) that have not been distorted by postmortem diagenetic effects are morphologically variable, and are not clearly distinguishable morphometrically as a group from non-avian coelurosaurian teeth, such as the small teeth of *Richardoestesia* [25], and the teeth of other stemward theropods [25, 27, 38]. Ornithurine teeth seem only marginally wider, relative to crown height, than *Richardoestesia* teeth for instance.

### Microstructure

#### Hesperornis

**Fig. 6:**
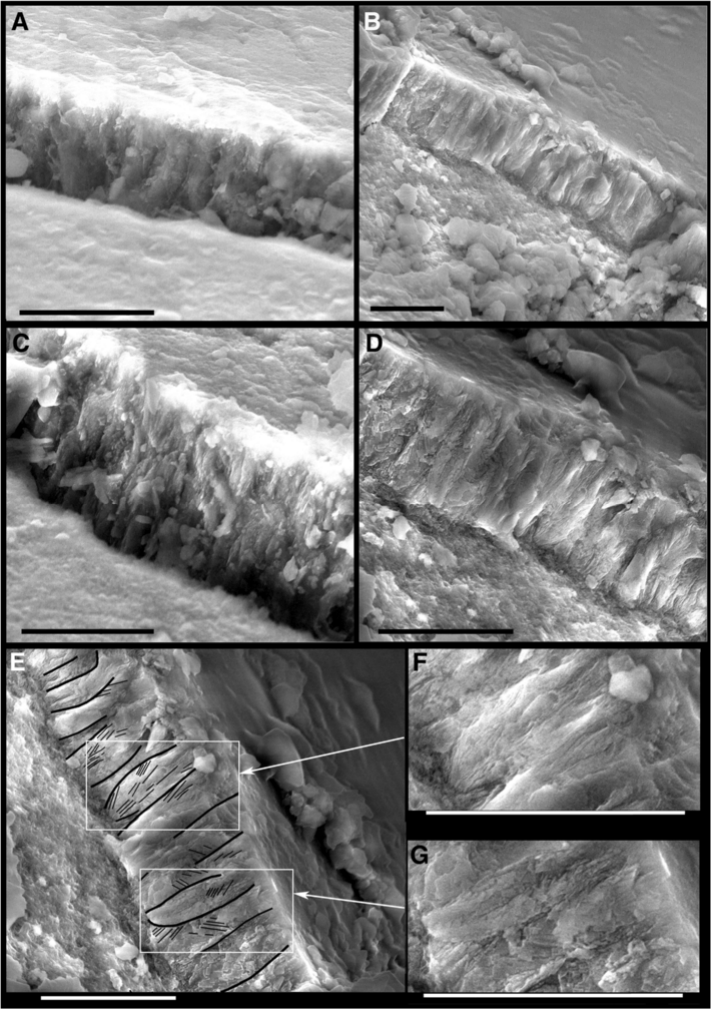
Enamel microstructure of the teeth of *Hesperornis* and *Ichthyornis*. All SEM micrographs. Low-angle views show sections of enamel down to EDJ, visible due to diagenetic fissuring and breakage. No etching was performed. These views reveal the thin Basal Unit Layer formed by parallel columns (of near-polygonal section) orthogonal to the EDJ and enamel surface. Each column is formed by assembled divergent parallel crystallites. **a**, **c** *Hesperornis regalis* (isolated tooth YPM.1206.**b**). **b d e f g** *Ichthyornis dispar* (third tooth in YPM.1775). in **e**, thick black lines are drawn to highlight some columns, whereas thin black lines highlight some of the divergent crystallites forming a column. **f**, **g** magnifications of regions in **e**. Crystallites are not all obvious since the surface has undergone no etching. Scale bars 5 μm

The enamel layer in *Hesperornis* (Fig. 6, Additional files 3 and 4: Figs. S2, S8) is extremely thin on the sides near the crown base (minimum ~4 μm). It increases progressively in thickness toward the apex, reaching ~10 to 15 μm at mid-crown height away from ridges and carinae (cf. below). Enamel thickness continues to increase up to the apex, at which point it reaches ~20 μm on the sides (orthogonal to enamel surface plane) (Additional file 4: Fig. S8). Owing to the value and rarity of these fossils, Scanning Electron Microscopy (SEM) observations were made without preliminary etching, on natural cracks in the enamel layer. Nevertheless, elements of enamel structure are discernible (Fig. 6). The enamel microstructure appears to be simple, composed of parallel columns orthogonal to the enamel-dentine junction (EDJ) and to the surface. The columns seem polygonal, 1 to 2 μm diameter, and the enamel layer they form appears to consist of a basal unit layer (BUL) *sensu* [17] (Fig. 6a, c). Each of the columns is formed of assembled crystallites that appear to be divergent within the column toward the surface, as in *Ichthyornis* (see below and Fig. 6e–g). No enamel tubules (*sensu* [29]) are observed (Additional file 3: Fig. S2).

The mesial and distal carinae consist of both (i) deformation (folding) of the enamel layer, implying that the EDJ is sub-parallel to the enamel surface, and (ii) moderate thickening of enamel; the ridges forming the fluted enamel ornamentation consist essentially of enamel thickening (Additional file 4: Fig. S8).

#### Ichthyornis

The enamel layer in *Ichthyornis* (Fig. 6, Additional file 4: Fig. S8) is as thin as in *Hesperornis* on the sides of the crown (~4 μm at crown mid-height), and it becomes thicker along the mesial and distal carinae (up to ~12 μm at crown mid-height), and towards the apex (up to 25–30 μm) (Additional file 4: Fig. S8B). The mesial and distal carinae are generated both by deformation of the EDJ, combined with moderate thickening of enamel to form the blades (Additional file 4: Fig. S8) as for *Hesperornis* carinae. Like in *Hesperornis*, the enamel structure of *Ichthyornis* is simple and composed of a BUL, the units of which are columns apparently polygonal in section, 1 to 2 μm in diameter, and orthogonal to both the EDJ and enamel surface. Each column appears to be composed of crystallites that are diverging in the direction of the enamel surface (Fig. 6b, d, e–g). The dentine in the *Ichthyornis* teeth is often altered by post-mortem alteration, such as microscopic perforations of presumed bacterial origin (e.g., in YPM.1460, as well as in the Maastricht tooth NHMM/RD271; Additional file 5: Fig. S7). This is an impediment for observing dentine structure (as well as dentine growth lines in sections; see below).

##### Maastrichtian ornithurine tooth

The ornithurine tooth NHMM/RD271 lacks enamel over the basal ~1/3 of the crown. Enamel thickness (Additional files 5 and 4: Figs. S7, S8) increases towards the apex, and reaches, at ¾ of crown height, ~3.5 μm on labial or lingual faces, away from the mesial and distal carinae, and 15–20 μm at the carinae. Enamel thickness is maximal at the apex, at ~45 μm (measured orthogonal to surface) (Additional file 4: Fig. S8). The carinae consist of moderately thickened enamel, combined with deformation of the EDJ, as in *Ichthyornis* (Additional file 5 and 4: Fig. S7B, S8A). Presumed microbial action has altered the dentine in a similar fashion as seen in several *Ichthyornis* teeth, more prominently just below the enamel layer (Fig. 1i, Additional file 5: Fig. S7).

### Tooth implantation and replacement

#### Hesperornis

The teeth of *Hesperornis* are implanted in a groove, as is visible in the dentary fragment YPM.1206A (Fig. 2). In this specimen, the groove preserves ten labio-lingual constrictions, which narrow the groove width at regular intervals. Each of the three preserved *in situ* teeth are in the groove between two consecutive constrictions (Fig. 2b). The largest tooth, T_H2_, is displaced due to postmortem disturbance: half of the small root portion preserved emerges from the dentary, and the whole tooth is displaced distally and dorsally (away from the groove). T_H1_ and T_H3_ are in their original biological position at time of death. In addition, an embryonic tooth crown apex (T_H4_) is present under T_H2_, and parts of a tooth (root portions and some cementum T_HC_) are present behind T_H3_ (Fig. 2a). In the more distal part of the dentary fragment, another tooth (turned upside down) is located deeply in the groove, attesting to strong post-depositional displacement. A single, empty alveolus is present at the mesial end of the dentary. The constrictions within the groove delineate each of the tooth positions. In transverse section these constrictions appear to be bulges of the main dentary bone from mid-depth to the bottom of the groove (Fig. 2: compare panels e and f). Each constriction therefore consists of a ridge from one edge of the groove to the other edge, through the bottom. The constrictions delimiting tooth spaces are entirely enclosed only at their bases (Additional file 6: Fig. S6). They are formed of the same bone tissue as the rest of the dentary, and are not secondarily deposited (Fig. 2g).

**Fig. 7:**
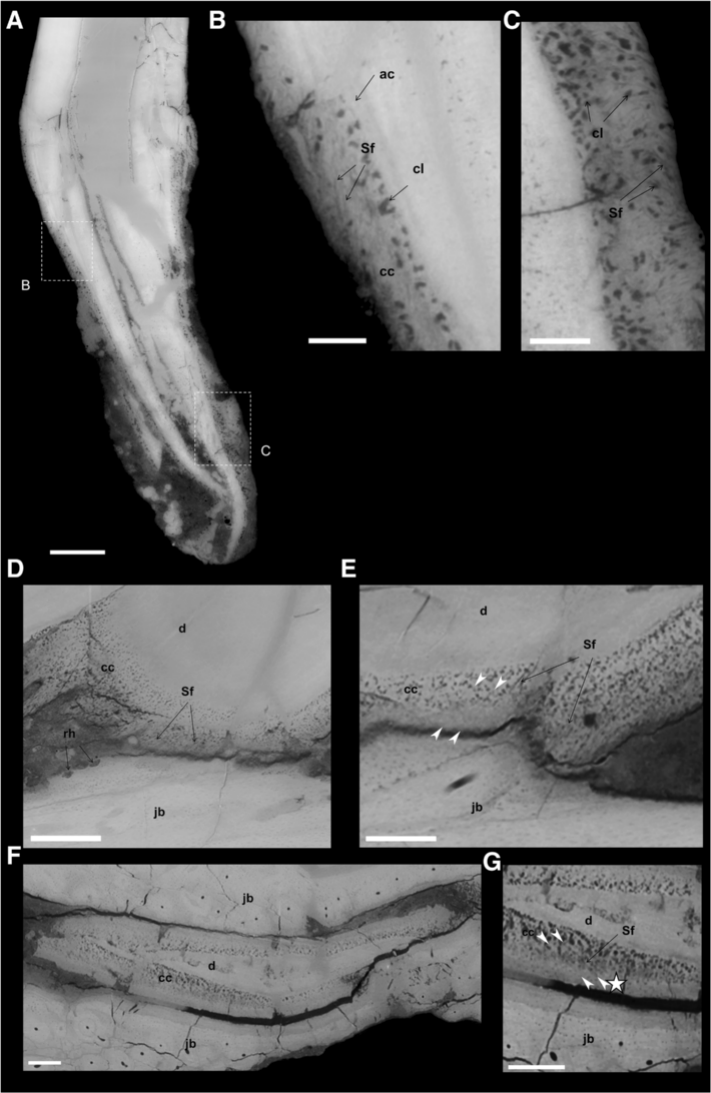
Synchrotron x-ray microtomographic images showing *Hesperornis* tooth implantation and replacement. **a** Basal-apical, transverse section of *Hesperornis regalis* isolated tooth YPM.1206.B showing the cementum and tooth attachment tissue on the root. Insert **b** at labial edge of the tooth root with the different cementum tissues: fine acellular cementum layer (ac) and the cellular cementum (cc) with large cementocyte lacunae (cl). Sharpey’s fibers (Sf) are also visible. Insert **c** at lingual edge of the tooth root showing cementum with numerous large cementocyte lacunae (cl) and Sharpey’s fibers (Sf). **d**–**g** Virtual sections of the tooth attachment in the dentary YPM.1206A. **d** Parasagittal section of the first tooth T_H1_ in YPM1206A. The tooth (dentine, d) is inserted via Sharpey’s fibers (Sf) into the jaw bone (jb). Some resorption holes (rh) confirm resorption of the jaw bone surrounding teeth during dental replacement. **e** Higher magnification of this region of implantation shows the cellular cementum directly attached to the jaw bone via Sharpey’s fibers. **f** Virtual transverse section through the fragmented tooth and cementum (T_Hc_) preserved in front of T_H3_ in the groove. The tooth is poorly preserved, with only some root dentine (d) still present. It is surrounded by the cementum. **g** Higher magnification on the cementum and jaw bone shows that the cementum is directly attached to bone tissue (asterisk) with Sharpey’s fibers. This tissue is separated by a space (possibly a diagenetic crack), from the rest of the groove bone wall, but it is histologically similar to this bone wall (with osteocyte lacunae). In **e** and **g** the white arrowheads point to the direction of visible Sharpey’s fibers that can be traced across the cementum-bone boundary. Scale bars **a** 0.5 mm, **b c** 0.1 mm, **d** 0.5 mm, **e f g** 0.25 mm

The isolated tooth YPM.1206B exhibits a substantial amount of preserved cementum (Fig. 7a). The external layer of cementum is cellular, and is separated from the dentine by a less obvious acellular cementum layer. The cellular cementum is thick and well developed, with large cementocyte lacunae, and some Sharpey’s fibers (Fig. 7b, c) (feasibility of Sharpey’s fibers imaging with synchrotron being demonstrated [39]). The cementocyte lacunae are quite abundant in comparison with other teeth covered in the present study (Additional file 7: Fig. S4). Teeth in the dentary YPM.1206A all preserve some cementum (Fig. 7d–g, Additional file 8: Fig. S5A). This cementum displays numerous Sharpey’s fibers, a condition for the presence of a periodontal ligament in life [34]. However, it appears that the cementum is very close to the adjacent dentary bone when *in situ*, with less than 50 μm separating them (Fig. 7e–g). This would have been the space occupied by a periodontal ligament, if indeed there was one. In places, the root cementum of one tooth (Fig. 7g) seems to be closely adjacent to a tissue (asterisk) similar to the dentary bone tissue, with Sharpey’s fibers again being present. Some individual Sharpey’s fibers can be traced across the cementum-bone limit (Fig. 7e, g), indicating the absence of a periodontal ligament, at least locally. Remodeling of the bone of the dentary is directly visible in YPM.1206A (Fig. 7d, Additional file 8: Fig. S5B-D). Resorption holes and lines are present in the dentary bone adjacent to the teeth, attesting to a similar process of resorption as observed for alligator alveolar bone [40], despite the fact that *Hesperornis* does not exhibit alveolar bone.

#### Ichthyornis

The *in situ* teeth within the dentary fragment YPM.1775 are inserted in sockets (Fig. 3a, b). Three teeth are preserved in the anterior part of the dentary. Two of these are not fully grown, and only beginning to erupt (T_I1_ and T_I3_), while the third represents a replacement tooth at a much earlier stage of development (T_I2_). As in *Hesperornis*, the teeth T_I2_ and T_I3_ are oriented with the crown pointing distad, whereas T_I1_ shows the opposite orientation, which seems to be due to post-mortem displacement. In the distalmost part of the specimen, the small apex of an incipient tooth crown is preserved on the side of one socket (Fig. 3c). It is oriented towards the labial side of the dentary, which is presumably due to postmortem displacement. The sockets in which the teeth are inserted are separated by alveolar bone septa. These septa are formed of primary woven bone tissue, with large lacunae, whereas the jaw bone itself is formed of lamellar bone tissue (Figs. 3d–f). The bone tissue of the septa is well vascularized.

**Fig. 8:**
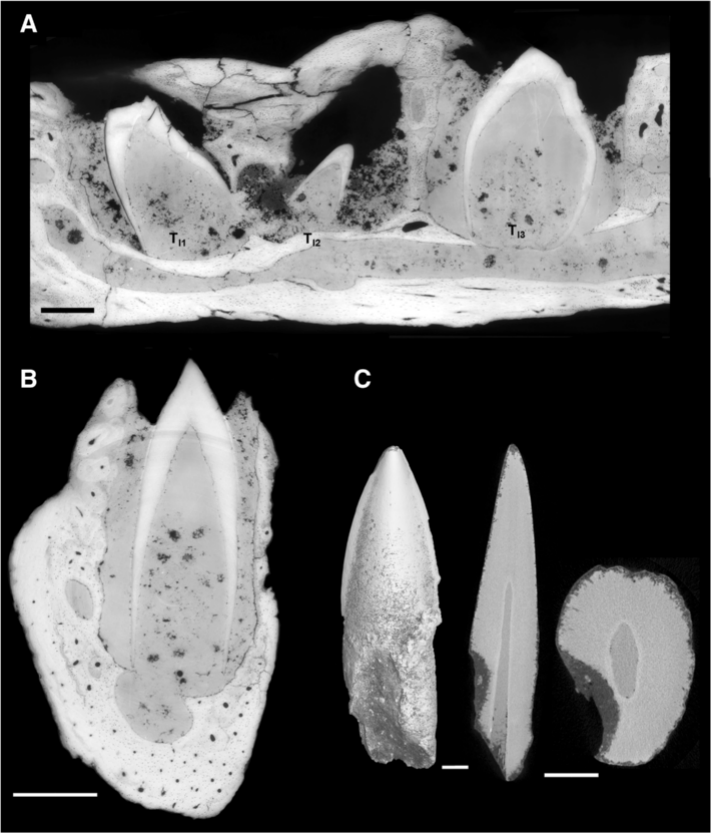
X-ray microtomographic images showing *Ichthyornis* tooth implantation and replacement. **a** Parasagittal view of the *Ichthyornis dispar* dentary fragment YPM.1775 with teeth inserted in sockets. The alveolar space is filled with sediment and there is no attachment tissue or preserved cementum around the root of the teeth. **b** Transverse section of the *Ichthyornis dispar* dentary with T_I3_ tooth. **c** *Ichthyornis dispar* isolated tooth (UAM_PV93.2.133_2): in lingual view, basal-apical tooth section and horizontal root section respectively from left to right, exhibiting the resorption pit. The root is resorbed there in the form of an oval scar by the new replacement tooth that was growing lingually and migrating into the functional tooth root. **a**, **b** Synchrotron x-ray microtomography, **c** conventional x-ray microtomography. Scale bars 0.5 mm

The isolated *Ichthyornis* teeth do not preserve any attachment tissue or cementum, which is also due to the fact that the specimens in question, UAM_PV93.2.133_1 and UAM_PV93.2.133_2, do not preserve their entire roots, and YPM.1460 consists only of the crown apex (Fig. 1c, d, e). Similarly, the teeth implanted in the dentary of YPM.1775 do not exhibit any attachment tissue (Fig. 8a), consistent with the incompletely grown and erupted state of these teeth. The alveoli are filled with sediment (Fig. 8b). Hence, the preservational state and/or developmental stage of the *Ichthyornis* specimens do not allow clear testing of the presence or absence of Sharpey’s fibers and cementum.

A resorption pit is present on the lingual side of the root of the UAM_PV93.2.133_2 tooth (Fig. 8c). The root as preserved is not closed and the pit is open basally.

##### Isolated comparative teeth

Two comparative theropod teeth preserve the root in good condition, allowing assessment of some attachment tissues (Additional file 7: Fig. S4). The tooth TMP 1989.103.0025, presumably of the non-avian theropod *Richardoestesia isosceles* (see Discussion-identifications) shows both cellular and acellular cementum (Additional file 7: Fig. S4). Between the dentine and the apparently acellular cementum is a layer with a high density of relatively large lacunae. It is unclear whether the external layer of dentine encompasses the large lacunae, or whether these represent a layer of highly cellular cementum. Nevertheless, the tooth exhibits two types of cementum, as well as numerous Sharpey’s fibers (Additional file 7: Fig. S4). The tooth TMP 1996.012.0040, which putatively belongs to Aves (see Discussion-identifications), exhibits cementum as well. It also shows Sharpey’s fibers. *Hesperornis*, *Richardoestesia* and the tooth indet. all show well developed cementum. The Sharpey’s fibers exhibit different orientations in the *Hesperornis* tooth (fibers slanting basally at a very low angle to the cementum surface) vs. the two other teeth (fibers near-orthogonal to the cementum surface) (Additional file 7: Fig. S4).

### Tooth growth and development

**Fig. 9:**
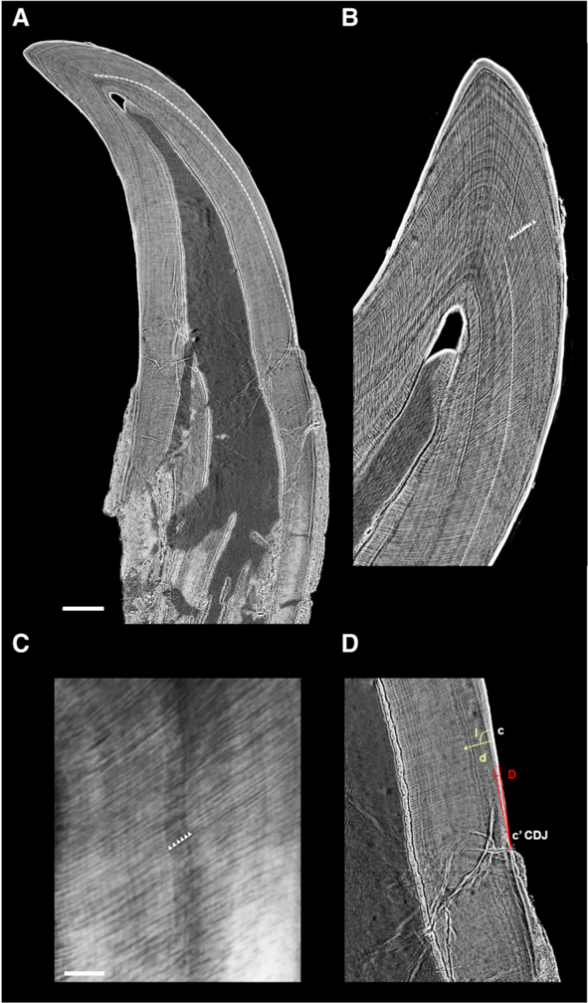
Synchrotron x-ray microtomographic images of *Hesperornis* tooth dentine increment lines (YPM.1206.B). **a** Parasagittal basal-apical section of the *Hesperornis regalis* isolated tooth YPM.1206.B, showing its dentine increment lines. **b** Higher magnification of the apex of the tooth from the same section. Regular incremental lines are observed throughout the dentine (white arrows), and are interpreted as daily lines. They are counted from the dentine horn to the incremental line corresponding to crown completion (dashed line). **c** Higher magnification of the series of incremental lines observed in the dentine and interpreted as daily increments. **d** Higher magnification at the cervix level of the area near the cementum-dentine junction (CDJ) in the root, for quantifying the tooth root extension rate. d, direction of the daily rate of dentine secretion (DSR); angle I, angle the dentine tubules make with the root surface; angle D, angle between an incremental line and the root surface. Scale bars **a** 0.5 mm, **c** 0.05 mm

The dentine increment lines observed in *Hesperornis* exhibit very thin intervals, around 3 μm (see Table 1; DSR daily secretion rate). Roughly similar values are observed in the other teeth (around 3.0 to 3.5 μm). These spacings are similar to those measured in human, pig and non-human primates —macaque, gibbon, orang-utan (~4.0 μm) (e.g., [41–44]). These short lines correspond to Von Ebner lines (Fig. 9). They represent daily increments of growth of the tooth [42]. Measurement of the spacing of these short period lines is an indication of rates of dentine formation [42, 45, 46]. We estimate the mean rates of tooth formation in *Hesperornis* and some comparative isolated teeth, in cases where preservation quality enables observation of dentine increment (Fig. 9, Additional file 9: Fig. S9, Table 1). Counts for *Hesperornis* indicate that it took 66 days to form a tooth. The tooth TMP 1986.052.0054 shows a rather similar formation rate of 62 days. The root extension rate calculated for *Hesperornis* is 48.2 μm/day. It is up to twice as high as that of isolated non-avian theropod teeth (from 24 to 31 μm/day; Table 1).

**Table 1:** Parameters of tooth growth measured and calculated from dentine increment lines in sections of teeth of *Hesperornis* and comparative specimens

	YPM.1206.B	TMP	TMP	TMP
	*Hesperornis regalis*	1989.103.0025 *Richardoestesia isosceles*	1986.052.0054 “Aves indet.”	1986.030.0039 “Aves indet.”
Mean tooth formation duration (days)	66	74	62	88
Angle I (°)	91.6	95.46	100.23	107.3
Angle D (°)	3.68	7.16	8.4	5.6
d = DSR (daily secretion rate) (μm/day)	3.1	3.5	3.5	3.1
Tooth extension rate at the cervix level (μm/day)	48.2	28.06	23.9	31.1

## Discussion

### Morphology and microstructure

Previous authors have noticed that the more mesially located teeth in the jaws of *Hesperornis* and *Ichthyornis* are progressively more recurved than the distal ones [6, 13, 47, 48]. This characteristic is shared by other toothed birds including *Archaeopteryx* [13, 49]. Hence, the isolated *Hesperornis* tooth YPM.1206B, which is highly recurved, is likely to derive from a mesial position within the jaw partly represented by the YPM.1206 specimen. Therefore, this isolated tooth must belong to a mesial portion of a dentary, since the premaxilla is edentulous in *Hesperornis* [6]. Furthermore, the tooth YPM.1206B must belong to the right dentary, i.e., precisely, YPM.1206A, judging from the lingual inclination toward the left side, visible in this tooth. *Ichthyornis* also exhibits toothless premaxillae [50]; this feature has been interpreted as illustrating partial dental reduction near the origin of the avian crown clade [4]. The predentaries present in *Hesperornis* and *Ichthyornis* (mesial to the dentaries) are also toothless [51, 52]. The mesial-distal gradient of tooth straightness in Ornithurae (Hesperornithiformes and Ichthyornithiformes) might reflect an adaptation to piscivory in both taxa. Conversely, in *Richardoestesia gilmorei* (which otherwise presents some dental similarities to toothed birds, but whose phylogenetic position within Coelurosauria is unclear) exhibits the opposite condition: the teeth become mesially straighter, and distally more recurved distad, possibly reflecting an adaptation to feeding “on insects and other soft-bodied prey” ([24]: 123). However, links between the mesial-distal gradient of tooth straightness and diet are far from obvious; for example, certain terrestrial predators (probably largely insectivorous) such as *Compsognathus* exhibit the same gradient [53] as the piscivorous ornithurines described here.

Our observations confirm and precise important differences in shape between *Ichthyornis* and *Hesperornis* tooth crowns, described previously [6, 47]. In *Ichthyornis* the crown bears very prominent ridges, mesially and distally, reaching the apex of the tooth; these ridges are sharp, cutting edges without serrations. In *Hesperornis*, mesial and distal ridges without serrations are present, but faint and far less developed than in *Ichthyornis*, and they do not reach either the apex or the neck of the tooth. In the more curved *Hesperornis* teeth, the distal ridge is shifted labially. At comparable mesio-distal positions along the jaw, tooth crowns are more constricted labio-lingually in *Ichthyornis* than in *Hesperornis*. The curvature of teeth in *Hesperornis* is distributed evenly, and affects the entire rounded crown (especially in mesial teeth). In *Ichthyornis*, the more mesially situated teeth are ‘recurved’ distad, but this results exclusively from a sharp angle between the root and the crown. The crown itself is therefore directed distad, but it is essentially straight. Finally, *Hesperornis* crowns bear fine basal-apical ridges (“fluted ornamentation” [17]), whereas *Ichthyornis* crowns are devoid of ornamentation. Confirming Sander’s observations on a presumed *Hesperornis* sp. tooth [17], we show that the fluted ornamentation of *Hesperornis* teeth are largely the product of thickening of the enamel layer, with no substantial visible EDJ preformation. The slight mesial and distal carinae of *Hesperornis* consist of a combination of moderate EDJ deformation, and moderate enamel thickening (the latter initially highlighted by O.C. Marsh [6]); enamel thickening is also observed towards the apex of the crown. In *Ichthyornis* as well as in the Maastrichtian tooth hereafter assigned to the Ichthyornithiformes, the well-marked mesial and distal carinae are also formed by a combination of EDJ shaping and enamel thickening.

The enamel of *Hesperornis* and *Ichthyornis* is prismless as in most non-mammalian amniotes (except agamid lizards). In both *Ichthyornis* and *Hesperornis*, the enamel consists of an extremely thin layer of columns orthogonal to the enamel surface, and each column is composed of divergent crystallites. The microstructure of the thin enamel layer (mostly <5 μm; thicker at the carinae and apex) therefore corresponds to one single basal unit layer (BUL), as suggested earlier on a presumed *Hesperornis* sp. tooth [17]. The microstructure of the enamel in both *Ichthyornis* and *Hesperornis* is highly simplified compared to that of the non-avian theropods illustrated by S.H. Hwang [28, 29]. This author also described the Schmelzmuster of enamel of two teeth of indeterminate Late Cretaceous birds [29], and compared them with that of the presumed *Hesperornis* sp. tooth [17]. Hwang interpreted a second, thinner layer over the BUL, composed of parallel crystallites [29]. Specifically, the second tooth examined was said to bear resemblance to *Hesperornis* as well [29], and its features were discussed in comparison with those of Sander [17] as if it was positively *Hesperornis*. However, we see no reason to consider that such a taxonomic assignment is supported. Hence, we interpret the lack of a second layer on top of the BUL in our specimens, as well as in the cf. *Hesperornis* sp. tooth [17] as a *Hesperornis* characteristic, and consider the teeth described in [29] to derive from probable, indeterminate Mesozoic birds. A thin BUL is thus confirmed for *Hesperornis*, and shown for the first time in *Ichthyornis*.

*Contra* [29] with Aves indet., no tubules originating from the EDJ and extending to the surface are observed in micrographs or in virtual sections of the enamel of *Hesperornis*, nor *Ichthyornis*. It seems that the observed tubules [29] are artifacts caused by acid etching employed to enhance surface observations. In [29], there are no transverse sections illustrated that could show the tubules originating from the EDJ and running to the surface. The ‘tubules’, which seem to be holes, are only observed in longitudinal and oblique sections.

The thinness and simplification of enamel in *Hesperornis* and *Ichthyornis*, with one BUL only, are apparently unique among studied archosaurs, and indeed reptiles [17, 28, 29]. In *Ichthyornis* and *Hesperornis*, enamel thicknesses of 4 to 10 μm (away from such zones of thickening as apex and carinae) amount to 0.27–0.30 % of crown height, whereas in the Nile crocodile for instance, which exhibits similarly-shaped teeth associated to largely piscivorous function, this percentage is 1.0 to 1.3 %. If this attests to a tendency toward reduction of enamel cover among crownward ornithurines, it might be significant that the Maastricht tooth hereafter assigned to Ichthyornithiformes exhibits a greatly reduced enamel covering, with enamel entirely absent from the basal part of the crown. An evolutionary process of enamel reduction preceding tooth loss on the line to crown birds would be in line with these observations. As a hypothesis, it would imply that the inactivation of enamel protein genes [54] was not strictly a consequence of arrested tooth development, but was perhaps already incipient in some Late Cretaceous toothed ornithurines.

### Attachment and implantation

Some of the teeth preserved *in situ* in the *Hesperornis* and *Ichthyornis* dentaries are obviously displaced within the dentary, due to post-depositional processes (as noted in [6]). This is most obvious in several of the teeth within the *Hesperornis* dentary. Displacement of these teeth might have been favored by the teeth being situated in a groove, with only slight bone constrictions around each tooth root. In contrast, the teeth of *Ichthyornis* are set in discrete alveoli, and therefore were likely more firmly attached and less liable to become displaced in the face of post-depositional factors such as water infiltration, chemical processes of alteration, sediment compaction and deformation, and other diagenetic effects. However, *in situ* teeth within the *Ichthyornis* dentary also exhibit some post-mortem displacement, including the most mesially positioned among preserved tooth, which is slanted mesially. Incompletely erupted teeth in *Ichthyornis* tend to be more inclined (distad) than fully erupted ones, which are set more upright with growth [6, 47]. Here we observe the same phenomenon in *Hesperornis*, with *in situ* teeth that are not fully erupted and grown (see below). The decomposition of non-mineralized attachment tissues such as the periodontal ligament, as well as alteration processes affecting root tissues (most visible in *Ichthyornis*), are likely responsible for this displacement. In addition, diagenetic compression has affected several of the specimens (especially the dentaries), and tooth roots within the *Hesperornis* dentary are hence often fractured and slightly distorted. However, the features preserved at certain locations allow characterization of dental attachment and implantation in both taxa.

In *Hesperornis*, the groove constrictions are formed by the same bone tissue as the surrounding jaw bone; they are not secondarily deposited (Fig. 2e). The same applies to the formation of the septum distally separating the unique alveolus in the mesialmost part of the dentary from the groove. In contrast, true sockets are formed of primary alveolar bone, which is histologically different from the bone tissue comprising the dentary (see *Ichthyornis* below for more details). The histological tissue comprising the constrictions in *Hesperornis* is therefore different from that of the true sockets observed in crocodilians or *Ichthyornis* ([31, 47]; Fig. 3d, e, f; Additional file 10: Fig. S3). In places, bone adjacent to teeth in *Hesperornis* has been resorbed simultaneously with the shedding of the tooth, and new bone has been redeposited with the development of the new replacement tooth. This results in resorption lines and holes (see Fig. 7d, f, Additional file 8: Fig. S5). The process of reformation of bone around the teeth is probably similar to that of true alveoli made of alveolar bone, yet we demonstrate the absence of true alveolar bone in *Hesperornis*.

The woven-fibred bone comprising the alveolar intersepta of *Ichthyornis* is similar to the juvenile caiman model described in [55] (see also Additional file 10: Fig. S3). Interdental septa are formed by the fusion of several bony plates running along the jaw. In adult specimens, these woven interseptal plates are remodelled and integrated into the jaw bone. Alveolar bone is composed of woven-fibred bone when growing quickly, or lamellar bone if growing at a slower rate [56–59]. The *Ichthyornis* alveolar bone attests to fast growth speed. It was formed simultaneously with the formation of the last series of teeth formed at death, since alveolar bone is principally a feature of the tooth root [34, 56]. During dental replacement, as a tooth is shed, alveolar bone is resorbed partially or totally and then reformed around the root of the new tooth (including transverse septa) by ectomesenchymal cells associated with the tooth [56]. In certain paravians such as troodontids, the interdental (alveolar) septa, where formed, are made of bone tissue that differs histologically from the laminar bone of the dentary [23]. The septa appear to be formed of true alveolar bone exhibiting woven texture with large lacunae, as in *Ichthyornis* or crocodilians.

‘Bone of attachment’ is an ill-defined term, which corresponds to bone tissue that is undifferentiable from alveolar bone, so it is synonymous ([34, 56]; *contra* [13, 36, 37]). The statement that so-called ‘bone of attachment’ in theropods would be attached to the tooth without a periodontal ligament and even without cementum [13], a form of ankylosis, is contrary to observations across archosaurs and other tetrapods [34]. Attachment of the root takes place via cementum attached to alveolar bone by a periodontal ligament (unmineralized to more or less mineralized). But periodontal ligament, if unmineralized, is not preserved in vertebrate fossils. And without fine-scale histological analyses of teeth in place in jaws, cementum can be overlooked, as it is often very thin in small archosaurs, and/or easily confused with dentine or bone.

Acrodonty, pleurodonty, subthecodonty, and thecodonty are terms describing gross morphology, but only histology analyses can properly differentiate among pertinent categories [56]. If these terms are practical descriptors of the depth of implantation, they are of limited interest in terms of phylogeny, because they appear highly subject to homoplasy; they are determined by the interplay of different amounts and arrangements of various attachment tissues, which can differ even within a single jaw [34]. True thecodonty has been known to characterize *a minima* archosaurs, mammals, mosasaurs (with a mineralized ligament) and some snakes (gomphosis, i.e., attachment through an unmineralized periodontal ligament) [34, 56, 60]. Attachment in thecodont snakes is hinged (i.e., the ligament is present on one side only; [56]). True thecodonty by gomphosis is furthermore exhibited by diadectids, which are early representatives of the amniote stem group, and its loss in some amniote groups appears to be secondary and derived from true thecodonty [34]. Acrodonty or pleurodonty (in extant squamates for instance) would therefore be secondary, and derived from thecodonty, again, not representative of the primitive state as previously thought [34]. Hence, thecodonty appears to be primitive for the amniote crown together with some stem amniotes (Cotylosauria), along with numerous associated characteristics: alveolar bone, cellular and acellular cementum, Sharpey’s fibers, lingual tooth replacement via the tooth germ entering the root through a resorption pit, and loss and resorption of most of attachment tissues including some alveolar bone during replacement. The longstanding restriction of thecodonty to crocodilians, mammals, marine reptiles, some Cretaceous snakes and certain dinosaurs is therefore obsolete [34].

Although the mode of tooth implantation in *Hesperornis* differs in some respects from classic thecodonty, the attachment mode is similar (despite the lack of alveolar bone). Implantation in a groove is presumably autapomorphic of *Hesperornis* (and possibly some other Hesperornithiformes). We propose that similar attachment attests to close homology, despite different implantation, of *Hesperornis* and typically thecodont taxa. *A contrario*, the superficially similar implantation in a socket of mosasaurs and crocodiles appears to be merely analogy, since the homologous attachment tissues involved exhibit very different tissue arrangements and amounts, and in mosasaurs the periodontal ligament is mineralized whereas crocodiles exhibit gomphosis [34].

The very thin space between root cementum and bone (locally < 50 μm) in *Hesperornis* could be interpreted as resulting from diagenetic compaction. Therefore, this space could have been wider in life, and could have accommodated a periodontal ligament. However, Sharpey’s fibers in the cementum of *Hesperornis* teeth can be traced in continuity through both cementum and adjacent bone. This indicates that a periodontal ligament was indeed absent and that cementum and bone were linked rather firmly via Sharpey’s fibers directly. Despite the absence of true alveolar bone, there is reworking of the dentary bone adjacent to the teeth. Hence, *Hesperornis* exhibits most features of thecodonty through gomphosis, albeit with secondary implantation in a groove. The thin spacing, and firm attachment via Sharpey’s fibers but without periodontal ligament, between cementum and bone might be an apomorphic, secondary feature compensating for a lack of sockets in maintaining firm tooth implantation in a groove. In *Hesperornis*, the cementum is well developed and rather thick, with a cellular layer and an acellular layer, comparable to the condition exhibited by crocodilians [16, 40], mammals [61, 62] and fossil marine reptiles [63, 64].

The presence of cementum has only rarely been observed in small non-mammalian amniotes [56], and has even been said to be unknown in non-avian theropods [65], making a supposed difference with birds. However, we report two types of thickened cementum at least in the non-avian theropod *Richardoestesia isosceles*. We therefore suspect that the perceived absence of cementum in non-avian theropods may reflect, firstly, the relative rarity of well preserved roots on isolated non-avian theropod teeth, as well as the near absence of data on attachment tissues from fine-scale investigations. Isolated teeth of *Hesperornis*, *Richardoestesia*, and an indeterminate (archosaurian, presumably theropod) tooth of comparable size, all exhibit well-developed cementum, as well as Sharpey’s fibers distributed within the cementum. Lower density in the distribution of cementocyte lacunae in the latter, indeterminate isolated tooth might reflect a slower rate of deposition (the density of entombed cementocytes in cellular cementum is hypothesized to be proportional to the speed of tissue formation; [61]). These shared features indicate thecodonty comparable to that in birds and crocodilians, in non-avian theropods such as *Richardoestesia*. Comparable features include cementum (acellular and cellular) overlying the root dentine, and Sharpey’s fibers, possibly with a periodontal ligament attaching cementum to bone in life. Following [34], this condition represents thecodonty through gomphosis, since these isolated teeth fell out of the jaw and the roots became detached from the bone, *post-mortem*, without damaging any part of the cementum or dentine. It implies that the periodontal ligament was non- or slightly mineralized (gomphosis), as opposed to mineralized (‘ankylosis’, where teeth generally do not fall out *post-mortem* fully intact with roots). Interestingly, the different orientations of the Sharpey’s fibers in the *Hesperornis* tooth vs. the two other of these three isolated teeth (strongly oblique Sharpey’s fibers with a strong basal-apical component, and fibers diverging basally around the root, in *Hesperornis*) suggest differences in attachment. These differences are possibly linked with differences in the constraints undergone by the teeth in life, driven by diet and/or feeding mode.

The groove with constrictions in *Hesperornis* is different from the groove in the distal part of juvenile crocodilian jaws, which does not exhibit constrictions (Additional file 10: Fig. S3; [40]). Septa form mesio-distally during development in the crocodilians, and are formed of alveolar bone [55]. In contrast, the constrictions in *Hesperornis* are not composed of alveolar bone, but are simply outgrowths of the main jaw bone, as is the separation of the unique mesialmost small ‘alveolus’. The groove in *Hesperornis* only superficially resembles the state in juvenile crocodilians. Therefore, this original autapomorphy cannot be interpreted simply as a neotenic characteristic. The groove observed in [47] within the distal portion of some *Ichthyornis* dentaries (inferred to belong to juvenile individuals) is very similar to that of *Hesperornis*, and exhibits constrictions. In *Ichthyornis* the rest of the teeth are inserted in sockets (and in presumed adults all teeth reside in sockets). The attachment is thecodont, with alveolar bone and alveolar septa that appear to grow in a similar manner as in the juvenile crocodilians (e.g., [31]). The presence of the groove, the derived loss of alveolar septa, and loss of alveolar bone, appear to represent hesperornithiform autapomorphies. Indeed, uniquely in *Hesperornis*, the groove extends nearly along the whole dentary (to the exclusion of the small, single, mesialmost alveolus revealed here). This appears to be the adult condition, and indeed no evidence of juvenile remains is known. *Hesperornis* can therefore be viewed as having secondarily lost thecodonty. The mesialmost individual small alveolus in the *Hesperornis* dentary might be a remnant of the thecodont condition of *Hesperornis* ancestors, now formed in the absence of alveolar bone. The reacquisition of a near-complete groove at adult stage effectively resembles the opposite of the progression of alveoli during crocodilian development (a common extant model for all archosaurs). This ontogenetic progression is apparently visible in early ontogenetic representatives of *Ichthyornis*, exhibiting a groove with constrictions similar to the juvenile crocodilian condition. Hence, the evolution of the groove in *Hesperornis* would have taken a path reminiscent of a neotenic character, but leading to a groove non-strictly homologous with that of juvenile crocodilians or even *Ichthyornis*. As for non-avian theropods, it remains to be seen whether or not a comparable groove exists in juveniles, with septa forming mesio-distally later in development (as might be expected in the hypothesis that this pattern would be plesiomorphic at least for theropods). Yet another type of pseudo-groove is known in adult troodontids [23]. Constrictions mark the delimitations of spaces for tooth implantation, on the sides and bottom of the groove, as in *Hesperornis*. But contrary to other archosaurs, the groove in Troodontidae is progressively better defined mesially, whereas sockets are formed in the distal part of jaw. The opposite condition is exhibited in juvenile crocodilians and juvenile *Ichthyornis* —where septa form first mesially and later toward the distal end. Even in *Hesperornis*, the single small alveolus is located at the mesialmost portion of the tooth row, as opposed to the troodontid condition. The constrictions hence would be shared with the condition in some juvenile archosaurs (probably convergent), while the precise aspect, or position (and presumably ontogeny) of the groove would represent a troodontid autapomorphy. The mesial absence (or nearly so) of alveolar septa in troodontids is hypothetically related to the crowding of teeth in this region in this group relative to other theropods [23].

To summarize, *Ichthyornis* and *Hesperornis* are thecodont through gomphosis (despite the presence of a secondary groove in *Hesperornis*). In *Hesperornis*, the tooth root is covered by two types of thick cementum (alveolar and non-alveolar), which is attached to alveolar bone via Sharpey’s fibers but at least partly without a periodontal ligament. This is also the case (cementum, fibers) in some isolated comparative teeth from indeterminate Mesozoic birds or non-avian theropods. Even though the preservation state of *Ichthyornis* did not allow recognition of cementum and Sharpey’s fibers, alveolar bone delimiting true sockets is recognized.

### Tooth growth and replacement

The *in situ* teeth of both *Ichthyornis* and *Heperornis* lack a large portion of their root. We interpret this as suggesting that these teeth were not fully developed, since tooth growth starts from the crown apex and completes with the root growing in the basal direction. Obviously, (1) the size of functional teeth *in situ* with incomplete roots (even considering displacement) leaves no place for a root if virtually replaced at its original, life location at the time of death; (2) the teeth that have apparently been displaced the least are generally small, with crowns only beginning to emerge from the occlusal border of the dentary, indicating growing replacement tooth stage. The larger tooth in our studied *Hesperornis* dentary, for instance, was presumably at a more advanced growth stage, although it was still not fully erupted. At this growth stage, these replacement teeth exhibited fully formed crowns, but not yet fully formed roots, which grew later. Fully-grown teeth (fully functional or even about to be replaced) are not preserved in the *Hesperornis* dentary for a variety of possible taphonomic reasons. For instance, such teeth, more prominently protruding from the jaw, were possibly more prone to falling out following death, especially with roots starting to be resorbed in the groove without alveoli. Alternatively, mature teeth may have been more prone to shedding; this may have been the case if they were fully emerged and close to being replaced and expelled by replacement teeth growing within their root. In the *Ichthyornis* dentary fragment, similarly, even the larger teeth present are not fully grown; they are much smaller than their alveoli and are not yet erupted. Their roots were only at the initial stage of growth at time of death, and would have presumably grown further only with the crowns erupting into their functional position.

#### Geometry of replacement in Hesperornithiformes

In *Hesperornis*, an isolated functional tooth with a lingual, oval resorption pit in its root has been described [6]. This resorption pit includes a tooth germ in place. A similar condition has been described in *Parahesperornis* [13, 48], a close relative of *Hesperornis* [8]. Tooth germs are observed here in the *Hesperornis* dentary. These are slightly displaced post-mortem, but nevertheless are situated under larger teeth, or lingually inside the groove against the lingual groove wall. They exhibit the previously described geometry of dental replacement of *Parahesperornis* and *Hesperornis*. YPM.1206B seems to show a possible lingual resorption pit in the root (Fig. 1a), which again is in line with the previously characterized tooth replacement in the Hesperornithiformes, whereby the tooth germ enters the root lingually and then develops under the functional tooth.

#### Geometry of replacement in *Ichthyornis*

The resorption pit observed on the side of one *Ichthyornis* tooth (Figs. 1e and 8c) is the first identified in this taxon, and reveals a similar dental replacement geometry to that observed in *Hesperornis* [6], *Parahesperornis* [13, 36, 37, 48], *Archaeopteryx* [31], crocodilians (e.g., [40, 66–68]; Additional file 10: Fig. S3), and stemward non-avian theropods (see below). The alleged differences in replacement geometry between non-avian theropods and the other taxa cited in [13, 36, 37, 48] are disproven. On the contrary, in all of these groups dental replacement proceeds on the lingual side by a small, growing replacement tooth germ entering the root cavity of the functional tooth through a lingual resorption pit, which is due to the activity of odontoclasts. Later, the replacement tooth grows under the functional one and finally expells it. Our observations therefore contradict the view of supposed ‘vertical’ tooth replacement in *Ichthyornis* proposed in [9], where the tooth germ would purportedly enter the functional tooth root from under its base without forming a resorption pit (or scar). This assumption was apparently motivated by the prior lack of data regarding resorption pits or scars in *Ichthyornis*. However, not all teeth preserving roots illustrate resorption pits, even in *Hesperornis*. In fact, teeth with resorption pits are very scarce among ornithurine teeth preserving roots (both isolated and in place within jaws). By comparison, in juvenile crocodiles, nearly all functional teeth exhibit root resorption at the same time (Additional file 10: Fig. S3). The replacement rate diminishes with age in crocodiles [69], and we lack data on growth series of *Ichthyornis* and *Hesperornis*. But our observations suggest that in these birds the frequency of dental replacement might have been markedly lower than in Crocodylia. As a result, we suggest that the number of ornithurine tooth generations during life would have been correspondingly lower, as well. Marsh ([6]: 125) was the first to claim that vertical replacement would characterize both *Ichthyornis* and crocodilians, as opposed to the horizontal (lingual) replacement of *Hesperornis* and mosasaurs. However, our study illustrates that all of these taxa, as well as all archosaurs in general, display horizontal, lingual tooth replacement, including *Ichthyornis* (this study) as well as crocodilians (e.g., [66]). Tooth replacement becomes vertical once the tooth germ has acquired its position in the functional tooth root; however, the geometry of replacement setup indicates that it is most accurate to characterize this pattern as lingual replacement. There is presently no evidence to suggest that vertical replacement ever existed among archosaurs.

### Paleobiology

#### Diet

Piscivory has long been assumed for both Hesperornithiformes and Ichthyornithiformes, based on distally recurved/hooked or distally slanting crown shape (increasingly towards the mesial end), presumably adapted for holding fish and other slippery prey. Marine depositional settings where the fossils are found are in accordance with this interpretation, both for Hesperornithiformes (flightless, foot-propelled divers adapted to pursuing fish underwater) and *Ichthyornis* (volant marine birds that likely acquired prey at the water’s surface). The basal-apical crown ridges exhibited by *Hesperornis* are also observed in several presumably piscivorous marine reptiles, suggesting an association with a piscivorous diet [17]. However, the functional and adaptive significance of these ridges remains unclear. *Ichthyornis* teeth, with their sharp cutting carinae, may suggest that these birds used their teeth to cut pieces apart before ingesting, whereas *Hesperornis* presumably swallowed prey whole more generally.

#### Growth dynamics

Our analyses of von Ebner increment lines indicate that one *Hesperornis regalis* tooth formed in 66 days, to be compared to what was observed [20, 21] for a crocodilian (110 days for juvenile *Caiman*) or a large theropod (264 days for juvenile *Tyrannosaurus*). Smaller teeth (from smaller species) are formed and replaced more quickly than larger ones (from larger species), for instance in sauropod dinosaurs [35]. But this size effect alone does not seem to explain a faster rate of tooth growth in *Hesperornis regalis*, especially given that *Hesperornis* was an extremely large bird, compared with other stem birds (almost the size of a human). Moreover, as was noticed earlier [70], the increment lines observed in several studies have an anomalously large width (from 10 to roughly 20 μm in [20, 21], and 15 μm in [35]), and may in fact correspond to Andresen lines instead of daily increments (intervals between Andresen lines corresponding to several days). Thus, the relatively high estimates of tooth replacement rates based on those figures should be considered with caution.

The rate of crown and root extension in *Hesperornis* (calculated here at the cervix level) is especially rapid compared with non-avian teeth studied here, and previous studies; however, crown extension rates for reptiles were not provided in [20, 21]. The rate of human premolar tooth extension has been shown to be variable: it begins slowly (at a value of 4 μm/day), rises (to a maximal rate of 8 to 18 μm/day) and reduces again as the apex of the root closes [71]. Our extension rates, calculated at the cervix level, are higher than these and other primate tooth measurements (e.g., different *Proconsul* teeth yield values from 6 to 34 μm/day; [72]). High extension rates in *Hesperornis* teeth might correspond to a functional need for the rapid installation and anchorage of new teeth when preceding teeth had fallen out. In addition, elongated and thin teeth are functionally linked to piscivory, which might explain part of the rate of crown extension (crown height vs. width or length).

Our suggestion of a lower number and frequency of dental replacements in *Hesperornis* (see above in ‘tooth growth and replacement’) than in non-avian theropods or Crocodylia (see [24]) might be tested in future studies of dentine increment of functional teeth with associated replacement teeth, in ornithurines. Incidentally, based on bone histology, ornithurines such as *Hesperornis* and *Ichthyornis* have been shown to exhibit body growth rates and inferred metabolic levels similar to those of modern birds. These rates are comparable to those of eutherian mammals —also fully homeothermic— and higher than those of comparatively stemward birds and dinosaurs, extant crocodilians, and other archosaurs [4, 73–76]. If *Hesperornis* exhibited growth rates similar to those of modern birds, adult size might have been attained within the first several weeks or months following hatching. As such, fewer dental replacements in *Hesperornis* might be expected, since turnover in the dentition would not be necessary to accommodate any additional growth of the jaws. This situation would contrast with that exhibited by most polyphyodont reptiles, in which continuous dental replacement throughout life allows shed teeth to be replaced with slightly larger ones in order to accommodate the continuous, slow body growth typical of poikilothermic organisms such as crocodiles [22].

### Dental characters and bird origins

Several alleged characteristics of the dentitions of *Hesperornis* and *Ichthyornis* have been cited in support of a non-dinosaurian or a non-theropod origin of birds [13, 36, 37, 48]. According to these authors, as summarized by Feduccia ([37]: 79), these features include, for birds and differing from ‘typical’ theropods: peg-like teeth without ornamentation or serrations; distal teeth with expanded roots; ‘subthecodont insertion’, teeth developing in a groove and septa forming around roots later; dental replacement from lingual side of roots, then ‘vertical’; oval resorption pit closed at base, on lingual side of the root of teeth being replaced; attachment with cementum and periodontal ligament. Our new observations allow us to critically evaluate these features.

Some Troodontidae have been shown to display many alleged ‘avian’ dental features [23, 77, 78]. This has led authors to acknowledge that these troodontids share homologous dental characters with birds [37]. These features include, for some or all troodontids: peg-like teeth without serrations; teeth without ornamentation on enamel; large root; constriction between crown and root; oval resorption pit closed at tooth base and germ tooth growing inside root of functional tooth; and absence of lingual interdental plates. Incidentally, peg-like teeth are not especially widespread in birds, and numerous species exhibit other morphologies (tooth crowns that are, for example, recurved, or bulbous). Even within the same individual, peg-like teeth may occur distally, but mesially the teeth can become highly recurved, such as in *Hesperornis* and *Ichthyornis*. This feature is even observed in *Archaeopteryx* [79]. Beyond troodontids, however, all of the characters cited as discriminating birds (and troodontids) from ‘typical’ theropods fall into one of two categories: 1) characters once thought to be exclusively avian and now known in ‘typical’ theropods, or 2) characters once thought to be unknown in birds but now reported among their representatives.

#### Features described previously as ‘avian’ but now known in ‘typical’, non-avian theropods

Serrations on tooth crowns are absent in most teeth of *Compsognathus* (except the distalmost ones), as well as on some of the teeth in *Ornitholestes* and probably *Coelurus* [80]. Serrations are also lacking in some troodontids such as *Byronosaurus jaffei* (including juveniles), *Mei long*, *Archaeornithoides deinosauriscus*, and *Urbacodon itemirensis* [81–86], as well as *Anchiornis* —which is either avian or troodontid— [78]. Serrations are lacking similarly on the teeth of *Buitreraptor* and *Rahonavis* (unenlagiine dromaeosaurs; [87, 88]), *Sinornithosaurus milleni* and *Microraptor* (microraptorine dromaeosaurs; [77]), dromaeosaurid hatchlings [89], probable deinonychosaurian teeth of uncertain affinity such as some “*Paronychodon*” types [90], as well as some Alvarezsauridae (*Shuvuuia*, *Mononykus*; [91, 92]), ornithomimosaurs, and therizinosaurs [87, 88]. Constriction between crown and root exists in some members of the Troodontidae such as *Troodon*, *Saurornithoides mongoliensis* and *Byronosaurus jaffei* [23, 77, 82, 83, 85, 93], *Microraptor* (Dromaeosauridae; [94, 95]), Alvarezsauridae (*Shuvuuia*, *Mononykus*) and some ornithomimosaurs and therizinosaurs [77, 87, 88, 91, 92]. Constriction between crown and root has been considered to be a derived condition within theropods [23]. Furthermore, absence of denticles and the constriction between the crown and the root are symplesiomorphic characters of the Maniraptoriformes and Maniraptora [87, 88]. Under this scenario, the denticles and absence of constriction in most dromaeosaurids, for instance, would be considered to be apomorphic. Incidentally, in birds with well-known dentitions (e.g., certain ornithurines and *Archaeopteryx*), constriction between the crown and root is not always pronounced; it is most prominent in distally positioned teeth (which tend to be straighter), and becomes faint to absent for mesial teeth (which tend to be more recurved, or slanted). Absence of interdental plates is probably a derived condition within theropods [23]. Interdental plates are absent in troodontids [23], which show jaw edges that are broadly similar to those of crownward toothed birds such as the ornithurines examined here. Expanded tooth roots are another supposedly ‘avian’ feature, but these are known in a number of non-avian theropods [23, 24, 65]. Taphonomic biases are certainly responsible for previously obfuscating the prevalence of expanded tooth roots in these stemward, non-avian theropods since, in the majority of cases, theropod teeth are found isolated, without most of the root. However, the same holds true for the fossil teeth of birds; these are often preserved in isolation without their roots (see many of the Aves indet. isolated teeth studied or cited here). In birds and some troodontids, the root has been described as being covered with cementum and held in place by a periodontal ligament. Post-mortem decomposition of the ligament would therefore cause the tooth to become detached. In ‘typical’ theropods, the teeth have been described as attached directly to cancellous bone designated under the term ‘bone of attachment’, via ‘sub-pleurodonty’ [13, 36, 37]. This would strengthen the root implantation after decay, and only the crown would therefore be expected to break off, and, hence, be found preferentially as isolated fossil remains. But attachment through cementum and periodontal ligament is widespread, including in dinosaurs, which are fully thecodont, not subpleurodont, and cancellous ‘bone of attachment’ is synonymous with alveolar bone (see Discussion-Implantation, attachment). The strength of tooth implantation in most avian and non-avian theropod taxa is likely comparable, since all exhibit a similar thecodont attachment with cementum, periodontal ligaments and alveolar bone.

We find the claim that isolated fossil bird teeth, in contrast to those from non-avian dinosaurs, should be expected to preferentially preserve the root (and that this would reflect a different mode of implantation and attachment; [13]) to be unfounded. The few known ‘isolated’ avian teeth that have been diagnosed to species (the material belonging to the Hesperornithiformes, Ichthyornithiformes, and *Archaeopteryx* discussed by these authors) are teeth that fell out of the jaw after death, and were generally found in close association with other cranial remains. These associations enable species-level identifications. However, a number of isolated bird teeth are known in the same state of preservation as isolated non-avian theropod teeth from a number of Mesozoic localities (e.g., Judith River Fm.). Unfortunately, the difficulty of positively identifying these to species casts doubt on the very identification of these remains as bird teeth. Hence, identification biases may help explain the false impression that the ratio of teeth preserved with and without roots differs between birds and ‘typical’ theropods. Conversely, a few typical isolated theropod teeth with their roots do exist in the published record; they have generally undergone little transport compared with shed teeth [96], but are otherwise preserved as well as shed teeth in proportion to their respective numbers produced until an individual’s death. Whereas isolated teeth bearing roots are teeth that have fallen out after death (due to decomposition of the periodontal ligament), isolated teeth without roots are shed during life, thanks to continuous replacement. Species with prolonged and/or frequent replacement also will naturally produce more shed teeth. They generally lack roots because the root is almost completely resorbed when a functional tooth is expelled by a growing replacement tooth, and because roots are rarely preserved in this manner. Continuous replacement yields a much greater quantity of shed teeth (all generations before death for a given individual) than the quantity one would expect for root-bearing teeth (originating from only one generation of teeth, at the time of death). The preservation state of avian vs. non-avian theropod teeth therefore provide no evidence pertaining to supposed differences in root attachment (*contra* [13]).

#### Features previously described as ‘non-avian’ but actually found in birds

As we highlight above, although isodonty has been cited as an avian feature, several birds exhibit highly recurved mesialmost teeth, and straight distalmost teeth, with a gradient in between. These taxa therefore qualify as exhibiting heterodonty. Describing avian teeth as peg-like is overly simplistic (see above); furthermore, we highlight the labio-lingual compression of avian teeth even in comparison with the non-avian coelurosaur *Richardoestesia*. These avian teeth do not qualify as peg-like, except possibly the distalmost teeth in heterodont taxa. Surface enamel ornamentation is often cited as a ‘non-avian’ feature, but is extremely well-marked in *Hesperornis* (‘fluted’ ornamentation, i.e., ridges of enamel), a fact often overlooked despite initially being described by Marsh [6]. More recently, well-marked basal-apical grooves were described in tooth crowns of an enantiornithine bird [97]. Serrations are cited as ‘non-avian’, but recently a Mesozoic enantiornithine bird from China has been shown to display tooth crowns with ‘crenulations’ (even though these differ somewhat from the serrations seen in many non-avian theropods in their shape and in their arrangement in two parallel basal-apical rows along the distal edge of tooth crowns) [98]. The alleged difference between a closed pit in birds and crocodilians, and a ‘scar’ open toward the basal direction of the root in theropods [13, 36, 37], is also not concordant with our observations, nor with the available, published data. In *Ichthyornis* we see a resorption pit that is ovoid, on the lingual side of the root, and open at the basal edge of the preserved root. Depending on the degree of resorption (and preservation) of the functional tooth root, and the degree of penetration of the tooth germ at time of death, there is a range of degrees of extension of resorption pits, even within a given species. Hence, *contra* [13], the resorption pit is not always closed at its base in birds, nor in Crocodylia (Additional file 10: Fig. S3). Conversely, a dromaeosaur tooth has been reported to exhibit a closed resorption pit at its root [65]. ‘Interdental plates’ are situated between teeth but lingual to the tooth row. Though superficially individualized, these plates are integrally part of the jaw bone, and are histologically continuous with the adjacent bone (be it the dentary, maxilla, or premaxilla; [99]; *contra* [13]; *contra* [36, 37]). Interdental plates are absent in *Hesperornis* and *Ichthyornis*; the lingual edge of the jaw maintaining the teeth is totally continuous with the rest of the jaw bone. *Archaeopteryx*, however, possesses interdental plates similar to those of most ‘typical’ theropods, consistent with its stemward position with respect to *Hesperornis* and *Ichthyornis* [49, 79]. This character, which is widespread and primitive among archosaurs, was independently lost several times throughout archosaur evolutionary history, perhaps through fusion and a smoothing of the grooves delimiting the plates in lingual view. Disparity in the number and degree of individualization of the plates indicates considerable plasticity across their evolutionary history, with possible occurrences of re-evolution of plate individualization.

#### ‘Vertical’ vs. ‘horizontal’ families of replacement teeth

*Contra* [6] and *contra* [9], *Ichthyornis* shows the same kind of lingual replacement as Hesperornithiformes, *Archaeopteryx*, some troodontids, and many ‘typical’ theropods (*contra* [37]). ‘Vertical’ replacement appears completely absent in archosaurs, whereas lingual replacement appears to be the rule. Another purported difference between the avian condition and that of ‘typical’ theropods was that, in the latter group, the replacement tooth would grow lingual to the functional tooth, without migrating within its root —at most making a scar in the lingual side of the root— before the shedding of the functional tooth [13, 36, 37]. This would represent a difference between non-avian theropods on the one hand, and birds and crocodilians on the other hand; in the latter two groups most of the growth of a replacement tooth, from germ stage to the stage where the functional tooth sheds, takes place inside the functional tooth root after having migrated there through a resorption pit created by odontoclasts around the tooth germ. Observations in one troodontid [77] indicate that the tooth germ makes an ovoid, closed resorption pit in the lingual side of the functional tooth root, and grows inside the latter root, which is considerably expanded. It seems that simultaneously the tooth germ makes a resorption hole in the bone wall (not observed in birds or crocodilians), a possible consequence of there being less space around the teeth in troodontids. The geometries of tooth replacement in birds and troodontids, vs. most other theropods, may be seen as the two ends of a continuum with many intermediate geometries not well documented due to the rarity of sufficiently well preserved series of replacement teeth. It is conceivable that the avian and crocodilian replacement geometries evolved from a plesiomorphic geometry retained by stemward theropods. Indeed, the typical theropod geometry is widely seen in other dinosaurs, such as sauropods with their batteries of replacement teeth lingual to functional ones [35]. Moreover, the ‘typical’ theropod condition is probably not uniform. Currie and Zhao [65] reported a drawing of a dromaeosaurid tooth with a closed ovoid resorption pit in the side of its expanded root. These authors ascribed the comparative rarity of such teeth in theropods as being due to tooth replacement occurring at a higher angle in relatively narrow jaws, resulting in that stage of replacement being “more transitory” ([65]: 2245).

An apparently real difference between many non-avian theropods, other (non-avian) dinosaurs, and archosaurs in general (e.g., [31]), vs. birds and some troodontids, consists of the existence in the former of variable ‘batteries’ of replacement tooth germs lingual to a functional tooth, whereas in birds (and some troodontids) a maximum of one replacement germ is hitherto observed lingual to a functional tooth or inside its root. Again, this decrease of the number of replacement teeth at a time is probably in line with a lower number and frequency of dental replacements (oligophyodonty) in birds in general, as already hypothesized in *Archaeopteryx* [31, 100].

To summarize, *Hesperornis* and *Ichthyornis*, the most crownward toothed birds known, show numerous derived dental features. These include extremely thin and simplified enamel in both taxa, and the presence of a groove housing the teeth in *Hesperornis*. Additionally, numerous features of their dental biology have been erroneously characterized in existing literature (ranging from geometry of dental replacement, form of resorption pit, and similarity of implantation between *Hesperornis* and *Ichthyornis*). We provide evidence for accurate assessment of these and other features. Furthermore, we demonstrate that many dental features do not radically differ between theropods and several other dinosaur groups, birds, and crocodilians (including geometry of dental replacement, and presence or not of interdental lingual plates). Indeed, the supposedly ‘avian’ condition appears to be much more phylogenetically widespread than previously reported. Arguments suggesting that the dentitions of *Hesperornis* and *Ichthyornis* provide evidence for a non-theropod, or non-dinosaurian origin of birds are therefore in error. Additionally, we confirm that some of the so-called ‘avian’ dental characteristics are only shared by certain non-avian theropod subclades, such as troodontids. These homologous characters add to the great phylogenetic proximity between troodontids and birds, now acknowledged to such an extent that one of these two clades is probably a subclade of the other [37, 101–103]. Finally, we contribute to document that some characters generally assumed to be absent in birds (e.g., ornamentation of the enamel, serrations) are occasionally present.

### Toward more precise identification of late cretaceous isolated avian teeth

Distinguishing morphologically between possible isolated avian teeth and *Richardoestesia* isolated teeth has already been shown to be difficult [25]. On a graph showing crown base width vs. height (Fig. 5), isolated TMP teeth studied here (Fig. 4) plot together with *Ichthyornis*, *Hesperornis* and other avian teeth studied, as well as the teeth identified as avian by Sankey et al. [25] (a sample that includes some of our TMP teeth; see Material and Methods): compared to *Richardoestesia* the avian tooth crowns seem to be proportionately larger at base, relative to their height; this trend is accentuated by the two larger of TMP teeth in our sample (Fig. 5). All of these isolated teeth are found in the same, or contemporaneous, localities in North America, where important numbers of such fossils are found in diverse localities [25]. Hence, it is useful to find further criteria for identification of isolated teeth as avian or non-avian. The two larger TMP teeth that also stand out in terms of their relative crown width vs. height, actually display additional characteristics, and altogether this questions their supposed avian status. One (TMP 1989.103.0025) bears a well-marked constriction between crown and root, and was re-identified recently as *Richardoestesia isosceles* (Coelurosauria *incertae sedis*; [27, 38]), *contra* [25]. The other large tooth (TMP 1996.012.0040) bears no clear constriction, is rather straight and bears no serrations, but exhibits a wear facet at the tip of the crown. We concur with [27, 38] that TMP 1989.103.0025, with its peculiar small serrations limited to a small part of both the mesial and distal carinae, does not belong to a bird. TMP 1996.012.0040 might be avian, but in the absence of associated skeletal remains, no precise identification is possible. Interestingly, wear facets are scarce, but not unknown in bird teeth (some are observed in presumably insectivorous birds, like *Archaeopteryx* [104]; or *Pengornis* [105]), despite the general inference that birds do not process food with their jaws [4]. We consider that TMP 1996.012.0040 is better assigned to cf. Aves indet. These two larger teeth might not belong with the avian teeth as they plot in a different portion of morphospace (Fig. 5). We consider that TMP 1989.103.25 is more likely to be compatible with *Richardoestesia isosceles* [27, 38] despite its marginal position, also relative to the other *Richardoestesia* teeth. In that case, the labio-lingual slight crown enlargement (relative to height) would appear to discriminate only marginally the ornithurine teeth from most contemporaneous non-avian North American theropod teeth. One TMP tooth crown (TMP 1986.052.0054) bears no serrations, and would fit well with birds on the basis of its ornithurine-like morphology; we concur with [25] in assigning it to Aves indet. Finally, two tooth crowns (TMP 1986.030.0039 and TMP 1994.031.0032) bear serrations recalling non-avian theropods. A single Mesozoic bird is now known to have crenulated teeth, reminiscent of serrations, albeit with unique characteristics of shape and distribution [98]. As a result, an avian status for those two serrated crowns is not totally excluded, but it is more likely that they belong to non-avian theropods. We consider it most appropriate that these teeth are referred to Theropoda indet. in the absence of associated remains.

The Maastricht tooth (Fig. 1h) is shown here to be positively identifiable as either belonging to *Ichthyornis* sp., or to a closely related taxon within the Ichthyornithiformes. It shows the characteristic triangular, labio-lingually compressed crown shape, with sharp, unserrated mesial and distal ridges, and very thin enamel (mostly around 5 μm in thickness). The whole preserved tooth shows the characteristic ichthyornithiform angle between crown and root (the root is visibly well developed despite the fact that it lacks most of its basal portion). The tooth size is also compatible with its diagnosis as *Ichthyornis*. The associated elements preserved alongside the tooth also allowed Dyke et al. [106] to recognise a closer affinity of the specimen with *Ichthyornis* than with any other known ornithurine taxon, despite the Maastricht specimen’s relatively larger size. Hence, we consider that an assignment of the specimen to the Ichthyornithiformes is pertinent. This represents the only record of Ichthyornithiformes outside North America, and the expanded distribution of this clade parallels that of the Holarctic Hesperornithiformes. This specimen not only represents one of the youngest known ichthyornithiforms [10], but indeed one of the youngest known non-neornithine ornithurines. The extension of the crownward-most portion of the avian stem towards the Cretaceous-Paleogene boundary supports the idea that the proximal avian stem survived until, and perished in, the end-Cretaceous mass extinction event [10, 107].

## Conclusions

Tooth morphology and ornamentation differ greatly between the Hesperornithiformes and Ichthyornithiformes. This suggests differences in the modes of feeding between these birds, *Ichthyornis* presumably cutting fish into pieces before ingestion, to the difference of *Hesperornis*. A tooth associated with postcranial remains from the Maastrichtian of Europe is the first Old World, and youngest record of the major Mesozoic clade Ichthyornithiformes. *Hesperornis* and *Ichthyornis* exhibit similar, extremely thin and simple enamel with BUL only. The extension rate of *Hesperornis* tooth dentine appears relatively high compared to non-avian dinosaurs. Root attachment is found to be fully thecodont via gomphosis in both taxa, but in *Hesperornis* secondary evolution led to teeth implantation in a groove, except for one mesialmost tooth dentary alveolus. This implantation in a groove, presumably less firm than in alveoli, seems to be compensated by firm root attachment, at least locally without a periodontal ligament. Dental replacement is shown to be lingual via a resorption pit in the root, in both taxa. There is no ‘vertical’ replacement, and no difference between *Hesperornis* and *Ichthyornis* in this regard. Our results allow comparison with other archosaurs and also mammals, with implications regarding dental character evolution across amniotes. Some dental features of the ‘last’ toothed birds can be interpreted as functional adaptations related to diet and mode of predation, while others appear to be products of their peculiar phylogenetic heritage. These observations highlight complexity in the evolutionary history of tooth reduction in the avian lineage and also clarify alleged avian dental characteristics, and the homologies (or not) with non-avian theropod teeth, in the frame of a long-standing debate on bird origins. Indeed, the supposedly ‘avian’ condition appears to be much more phylogenetically widespread than previously reported, now that it is better characterized in the details of shape, implantation, attachment, and geometry of replacement. Finally, new hypotheses emerge that will possibly be tested by further analyses of avian teeth, for instance regarding dental replacement rates, or simplification and thinning of enamel throughout the course of early avian evolution.

## Methods

### Institutional abbreviations

NHMM/RD, Natuurhistorisch Museum Maastricht, Maastricht, the Netherlands / Rudi Dortangs colln.; TMP, Tyrrell Museum of Paleontology, Drumheller, Canada; YPM, Yale Peabody Museum, New Haven, USA; UAM, Alabama Museum of Natural History, University of Alabama, Tuscaloosa, USA.

### Fossil material

For some of the less precisely identified materials, we denote previously published identifications with an asterisk (*). We propose new identifications for these specimens in the Discussion. The fossil specimens included in the present study are:

*Hesperornis regalis*: right mandible fragment with teeth YPM.1206.A, and isolated but associated tooth YPM.1206.B [6, 108]; *Ichthyornis dispar*: right mandible fragment with teeth YPM.1775, isolated tooth YPM.1460 (crown only) and tooth fragments YPM.1450 (see [6, 9]), and UAM_PV93.2.133_1 and UAM_PV93.2.133_2 (unpublished); Ornithurae indet.*: maxillary tooth NHMM/RD271 [106]; Aves indet.*: isolated teeth TMP 1996.012.0040 (unpublished), Aves indet.*: TMP 1986.030.0039 (crown only) (seems to be confused with TMP 1986.172.0053 of [25]), Aves indet.*: TMP 1994.031.0032 (crown only) (unpublished), Aves indet.: TMP 1986.052.0054 (crown only) [25]; *Richardoestesia isosceles* (Coelurosauria Incertae Sedis): TMP 1989.103.0025 ([27, 38]; was previously identified as bird by [25]).

### Extant crocodile specimen

Two one-year-old crocodile specimens (*Crocodylus niloticus*) from the Ferme aux Crocodiles (Pierrelatte, France) were scanned using conventional x-ray microtomography.

### Anatomical nomenclature

In toothless birds, rostral and caudal, and lateral and medial directions are generally used as descriptors of directions and orientations, including teeth positions, within jaws. Here, dealing with toothed birds, we adopt a more accurate nomenclatural system, used for toothed vertebrates: mesial and distal (equivalent to rostral and caudal) and labial and lingual (equivalent to lateral and medial). For teeth, length is mesio-distal, width is labio-lingual, and height is basal-apical. For slices in jaws and teeth, cutting planes are horizontal, parasagittal, or transverse (to jaw axis). As a result, parasagittal and transverse sections in a jaw across a tooth are also basal-apical with respect to the tooth axis.

### Provenance, geological context of the fossils

NHMM/RD271: Type Maastrichtian (SE Netherlands / NE Belgium), Maastricht Formation, base of Valkenburg Member (Late Cretaceous); biocalcarenitic limestone. TMP 1989.103.0025: Oldman Formation, middle Campanian, Pinhorn Ranch —Wendy’s Site, Alberta Canada; TMP 1994.031.0032: Scollard Formation, Late Maastrichtian, Griffith Farm, Alberta, Canada; TMP 1986.030.0039 and TMP 1996.012.0040: Dinosaur Park Formation, Late Campanian, Dinosaur Provincial Park, Alberta, Canada; TMP 1986.052.0054: Dinosaur Park Formation, Late Campanian, Steveville Railroad Grade, Alberta, Canada. UAM_PV93.2.133_1 and UAM_PV93.2.133_2: Mooreville Chalk Formation, Alabama, USA, early Santonian to early Campanian [109]. YPM.1206: Niobrara Formation, Kansas, USA. Late Santonian (~83 Ma); probable provenance from Goblin Hollow locality near Russell Springs, KS [110]. YPM.1450: Rooks County, Kansas, USA. Smoky Hill Chalk Member, Niobrara Formation. early Santonian [111]. YPM.1460: Twin Butte Creek [6], Niobrara Formation (Smoky Hill Chalk Member, early Santonian), Kansas, USA [9, 111]. YPM.1775: Niobrara Formation, Gove County, Kansas, USA [6]; i.e., 87–82 Ma.

### Synchrotron microtomography

All the samples scanned at the ESRF were imaged on the beamline ID19 using polychromatic beam and propagation phase contrast. Depending on the sample size and of the level of detail required, several setups were employed, yielding different levels of resolution. In all the cases, we used a FReLoN CCD 2 K14 camera (Fast Readout Low Noise; [112]), mounted on different optical magnification systems coupled to thin crystal scintillators to convert x-rays into visible light pictures. All the setup details used to scan these fossils are given in Table 2.

**Table 2:** Setup details of synchrotron beamline ID19 used to scan the fossil samples

Voxel size (μm)	1.28	3.5
Filters (mm)	Diamond 1.4 mm + Al 5.6 mm	Diamond 1.4 mm + Al 2 mm + Cu 0.5 mm
Insertion device	U17.6	U17.6
Gap (mm)	20	12
Scintillator	GGG:Eu 24.6 μm	GGG:Eu 47 μm
Refractive lenses	N.A.	N.A.
average detected energy (keV)	31	53
Machine filling mode	90 mA 16 bunches	200 mA 7/8 + 1 bunch
Propagation distance (mm)	200	250
Number of projections	4998 / 2499	5000
Half-acquisition mode	yes / no	yes
Exposure time (s)	0.3	0.15
Scan duration (min)	39	25 scans of 28 min each = 700 min
Sample list	TMP 1996.12.40 / TMP 1986.30.39 / TMP 1986.42.54 / TMP 1983.103.25 / TMP 1994.31.32 / YPM.1206B / YPM.1460 / YPM.1775	YPM.1206A

All the scans were reconstructed using a filtered-backprojection algorithm (software PyHST2, ESRF; [113]); each sample was processed both in edge detection mode [114], and with a single distance phase retrieval approach (modified from [39, 115]). This double reconstruction aimed at yielding data optimized for incremental growth line and other small detail visibility on one hand, and data for general 3D segmentation and larger structure contrast on the other hand.

After reconstruction, all the subscans of each sample were concatenated to generate a single stack of 16 bits TIF files. Ring artifacts were corrected on reconstructed slices [116], and the final stack was cropped in 3D close to the specimen to reduce the data size.

### Conventional microtomography

Conventional x-ray microtomography scans (except those of UAM_PV93.2.133_1 and UAM_PV93.2.133_2; see below) were performed at the Ecole Normale Supérieure de Lyon with a GE Phoenix Nanotom 180 device, and analyzed using VG-Studio MAX 2.2 software. UAM_PV93.2.133_1 and UAM_PV93.2.133_2 were scanned at the University of Texas High-Resolution x-ray CT Facility with an Xradia MicroXCT Scanner, and analyzed using VG-Studio MAX 2.2 software.

### Scanning electron microscopy

Scanning electron microscopy (SEM) experiment was carried out at the Université Lyon 1, on a Quanta 250 (FEI) SEM (JEOL), in low vacuum, at electron acceleration tension of 15.0 kV, with a working distance from 9 to 10 mm.

### Analysis of dentine incremental lines on virtual slices

The von Ebner lines in the dentine observed on virtual slices are counted in the primary dentine, from the base of the crown to the tip of the tooth (as with the enamel lines in [117]) (Fig. 7). Von Ebner lines form daily [42, 45]; spacings between ten consecutive lines are measured in the crown, allowing estimates of average values for the daily secretion rates (DSRs). The mean tooth formation duration of one *Hesperornis* specimen and three isolated, presumably non-avian teeth are estimated (Table 1).

The root extension method is applied as well [72, 118]. In this equation, c = d [(sin I/tan D)-cos I], d is the daily rate of dentine secretion (DSR), angle I is the angle the dentine tubules make with the root surface (or base of the crown), and angle D is the angle between an incremental or accentuated line and the root surface (Fig. 9, Additional file 9: Fig. S9).

It allows estimates of tooth extension rates at the level of the cervix (Table 1). For the tooth TMP 1986.030.0039, in which the root is not preserved, the measurements were made at the base of the crown (at the cervix level).

### Zoological nomenclature

We use the scientific name Aves —and the associated terms ‘avian’ and ‘bird’— to designate, and refer to representatives of the least inclusive clade comprising extant birds, *Archaeopteryx*, their most recent common ancestor, and all of its descendents. We follow in that the long-lasting, traditional usage (starting at least with Haeckel [119]) of naming this clade Aves (e.g., *The Zoological Record*), a practice followed by workers in accordance with compatibility with the Linnaean nomenclatural system. For specific references to the most recent common ancestor of all living birds and its descendents, we apply the term ‘crown bird’ —and crown avian. For specific references to taxa outside of crown bird diversity, but more closely related to birds than to crocodilians, we use the term ‘stem bird’ —and stem avian. This designation includes *Archaeopteryx*, Hesperornithes, and Ichthyornithes.

## Additional files

Additional file 1: Table S1. Morphometric parameters for the avian and non-avian teeth studied here and other, published specimens including *Richardoestesia* teeth (Sankey et al. 2002 [25], Hendrickx et al. 2015 [38]). (DOCX 40 kb) Additional file 2: Fig. S1. Supplementary morphometric comparisons of the studied teeth with other theropod specimens. (TIF 1082 kb) Additional file 3: Fig. S2. Enamel and dentine microstructure in *Hesperornis regalis. (TIF 3290 kb)*
Additional file 4: Fig. S8. Synchrotron and conventional x-ray microtomographic virtual sections in teeth of Ichthyornithiformes and Hesperornithiformes, illustrating variations in enamel thicknesses. (TIF 9506 kb) Additional file 5: Fig. S7. Post-mortem microbial attack visible on synchrotron x-ray microtomographic horizontal sections in teeth of Ichthyornithiformes. (TIF 1358 kb) Additional file 6: Fig. S6. Synchrotron x-ray microtomographic sections in the *Hesperornis* dentary YPM.1206A, showing aspects of the groove. (TIF 2336 kb) Additional file 7: Fig. S4. Synchrotron x-ray microtomographic views showing root cementum of *Hesperornis* and comparative teeth. (TIF 5049 kb) Additional file 8: Fig. S5. Synchrotron x-ray microtomographic views of *Hesperornis* tooth implantation and replacement. (TIF 7347 kb) Additional file 9: Fig. S9. Synchrotron x-ray micromographic virtual sections of comparative teeth, non-avian and presumably avian, showing dentine increment lines studied. (TIF 8021 kb) Additional file 10: Fig. S3. Conventional x-ray microtomographic views of juvenile crocodile tooth implantion and groove. (TIF 4109 kb)
