# Supplementary material for: Synchrotron imaging of dentition provides insights into the biology of Hesperornis and Ichthyornis, the “last” toothed birds
Source: BMC Evol Biol. 2016 Sep 23;16:178. doi: 10.1186/s12862-016-0753-6 (PMC5034473; doi:10.1186/s12862-016-0753-6)
Supplement: Additional file 1: Table S1. — Morphometric parameters for the avian and non-avian teeth studied here and other, published specimens including Richardoestesia teeth (Sankey et al. 2002 [25], Hendrickx et al. 2015 [38]). (DOCX 40 kb) [file 12862_2016_753_MOESM1_ESM.docx]

**Table S1** Morphometric parameters for the avian and non-avian teeth studied here and other, published specimens including *Richardoestesia* teeth (Sankey *et al.* 2002, Hendrickx *et al.* 2015).

| **Taxon** | **Specimen** | **Source** | **CBL** | **CBW** | **CH** | **AL** | **CBR** | **CHR** |
| --- | --- | --- | --- | --- | --- | --- | --- | --- |
| *Hesperornis regalis* | YPM.1206B | Present study | 0.90 | 0.71 | 1.32 | 1.68 | 0.79 | 1.47 |
| *Ichthyornis dispar* | UAM PV93.2.133_1 | Present study | 1.60 | 0.80 | 2.21 | 2.08 | 0.50 | 1.38 |
| *Ichthyornis dispar* | UAM PV93.2.133_2 | Present study | 1.61 | 0.90 | 2.78 | 2.86 | 0.56 | 1.73 |
| *Aves; cf. Aves | TMP 1996.012.0040 | Present study | 3.34 | 2.82 | 4.80 | 5.36 | 0.84 | 1.44 |
| *Aves; Theropoda indet. | TMP 1994.031.0032 | Present study | 2.20 | 1.10 | ? | ? | 0.50 | ? |
| *Aves; Theropoda indet. | TMP 1986.030.0039 | Present study | 2.07 | 0.89 | 2.53 | 2.89 | 0.43 | 1.22 |
| *Aves | TMP 1986.052.0054 | Present study | 2.39 | 0.83 | 4.50 | 4.60 | 0.35 | 1.88 |
| **Richardoestesia isosceles*; cf. *R. isosceles* | TMP 1989.103.0025 | Present study | 3.92 | 2.40 | 5.30 | 5.95 | 0.61 | 1.34 |
| Aves | TMP 1995.181.0060e | Sankey et al., 2002 | 1.40 | 0.60 | 2.40 | ? | 0.43 | 1,71 |
| Aves | TMP 1995.181.0060f | Sankey et al., 2002 | 1.90 | 0.70 | 2.50 | ? | 0.37 | 1.32 |
| Aves | TMP 1987.158.0077 | Sankey et al., 2002 | 1.90 | 1.90 | 3.10 | ? | 1.00 | 1.63 |
| Aves | TMP 1987.030.0010 | Sankey et al., 2002 | 2.00 | 1.00 | 3.70 | ? | 0.50 | 1.85 |
| Aves | TMP 1988.011.0065 | Sankey et al., 2002 | 1.90 | 1.80 | 2.90 | ? | 0.95 | 1.53 |
| Aves | TMP 1987.158.0076 | Sankey et al., 2002 | 2.40 | 0.80 | 3.30 | ? | 0.33 | 1.38 |
| Aves | TMP 1987.004.0046 | Sankey et al., 2002 | 1.90 | 1.00 | 3.60 | ? | 0.53 | 1.89 |
| Aves | TMP 1986.172.0053 | Sankey et al., 2002 | 1.90 | 0.70 | 2.90 | ? | 0.37 | 1.53 |
| Aves | TMP 1984.092.0205 | Sankey et al., 2002 | 2.00 | 0.70 | 2.70 | ? | 0.35 | 1.35 |
| Aves | TMP 1987.020.0008 | Sankey et al., 2002 | 2.20 | 1.00 | 4.30 | ? | 0.45 | 1.95 |
| Aves | TMP 1986.045.0027 | Sankey et al., 2002 | 1.60 | 0.90 | 3.70 | ? | 0.56 | 2.31 |
| Aves | TMP 1987.004.0019 | Sankey et al., 2002 | 3.00 | 1.00 | 5.50 | ? | 0.33 | 1.83 |
| Aves | TMP 1986.021.0068 | Sankey et al., 2002 | 2.00 | 1.00 | 3.70 | ? | 0.50 | 1.85 |
| Aves | TMP 1996.062.0051 | Sankey et al., 2002 | 1.60 | 0.70 | 3.20 | ? | 0.44 | 2.00 |
| Aves | TMP 1996.062.0062a | Sankey et al., 2002 | 2.20 | 0.90 | 3.80 | ? | 0.41 | 1.73 |
| Aves | TMP 1996.062.0062b | Sankey et al., 2002 | 1.70 | 0.80 | 2.70 | ? | 0.47 | 1.59 |
| Aves | TMP 1995.180.0049 | Sankey et al., 2002 | 1.80 | 0.70 | 3.20 | ? | 0.39 | 1.78 |
| Aves | TMP 1995.147.0030 | Sankey et al., 2002 | 1.90 | 0.70 | 2.40 | ? | 0.37 | 1.26 |
| Aves | TMP 1995.145.0034a | Sankey et al., 2002 | 1.50 | 0.60 | 2.10 | ? | 0.40 | 1.40 |
| Aves | TMP 1995.145.0034b | Sankey et al., 2002 | 2.10 | 1.00 | 4.00 | ? | 0.48 | 1.90 |
| Aves | TMP 1995.145.0034c | Sankey et al., 2002 | 2.00 | 0.80 | 3.50 | ? | 0.40 | 1.75 |
| Aves | TMP 1996.062.0062 | Sankey et al., 2002 | 1.70 | 0.80 | 3.50 | ? | 0.47 | 2.06 |
| Aves | TMP 1995.177.0079 | Sankey et al., 2002 | 1.90 | 0.70 | 3.00 | ? | 0.37 | 1.58 |
| Aves | TMP 1995.151.0021 | Sankey et al., 2002 | 1.50 | 0.80 | 2.30 | ? | 0.53 | 1.53 |
| Aves | TMP 1995.174.0052 | Sankey et al., 2002 | 1.80 | 0.70 | 2.30 | ? | 0.39 | 1.28 |
| Aves | TMP 1995.181.0010a | Sankey et al., 2002 | 1.80 | 0.90 | 3.10 | ? | 0.50 | 1.72 |
| Aves | TMP 1995.181.0010b | Sankey et al., 2002 | 1.80 | 0.80 | 3.30 | ? | 0.44 | 1.83 |
| Aves | TMP 1995.181.0010c | Sankey et al., 2002 | 2.20 | 0.80 | 3.30 | ? | 0.36 | 1.50 |
| Aves | TMP 1995.181.0010d | Sankey et al., 2002 | 1.60 | 0.80 | 3.30 | ? | 0.50 | 2.06 |
| *Richardoestesia* | TMP 1983.036.0233 | Sankey et al., 2002 | 4.70 | 2.30 | 12.30 | ? | 0.49 | 2.62 |
| *Richardoestesia* | TMP 1983.036.0242 | Sankey et al., 2002 | 3.70 | 1.60 | 8.30 | ? | 0.43 | 2.24 |
| *Richardoestesia* | TMP 1984.092.0268 | Sankey et al., 2002 | 3.50 | 1.50 | 5.50 | ? | 0.43 | 1.57 |
| *Richardoestesia* | TMP 1986.023.0090 | Sankey et al., 2002 | 4.00 | 1.70 | 10.00 | ? | 0.43 | 2.50 |
| *Richardoestesia* | TMP 1988.091.0028 | Sankey et al., 2002 | 2.90 | 1.00 | 5.30 | ? | 0.34 | 1.83 |
| *Richardoestesia* | TMP 1989.076.0063 | Sankey et al., 2002 | 2.80 | 0.90 | 5.20 | ? | 0.32 | 1.86 |
| *Richardoestesia* | TMP 1990.106.0006 | Sankey et al., 2002 | 1.70 | 0.70 | 4.00 | ? | 0.41 | 2.35 |
| *Richardoestesia* | TMP 1995.157.0029 | Sankey et al., 2002 | 1.40 | 0.70 | 2.80 | ? | 0.50 | 2.00 |
| *Richardoestesia* | TMP 1982.024.0078 | Longrich, 2008 | 3.41 | 1.30 | 4.92 | ? | 0.38 | 1.44 |
| *Richardoestesia* | TMP 1984.084.0247 | Longrich, 2008 | 3.14 | 1.50 | 6.13 | ? | 0.48 | 1.95 |
| *Richardoestesia* | TMP 1986.023.0105 | Longrich, 2008 | 2.21 | 1.16 | 4.80 | ? | 0.52 | 2.17 |
| *Richardoestesia* | TMP 1987.099.0048 | Longrich, 2008 | 2.00 | 0.99 | 5.35 | ? | 0.50 | 2.68 |
| *Richardoestesia* | TMP 1987.114.0005 | Longrich, 2008 | 2.83 | 1.08 | 4.43 | ? | 0.38 | 1.57 |
| *Richardoestesia* | TMP 1989.076.0083 | Longrich, 2008 | 2.87 | 1.21 | 3.35 | ? | 0.42 | 1.17 |
| *Richardoestesia* | TMP 1986.171.0009 | Longrich, 2008 | 2.11 | 1.16 | 3.75 | ? | 0.55 | 1.78 |
| *Richardoestesia* | TMP 1989.036.0355 | Sankey et al., 2002 | 3.80 | 2.00 | 10.00 | ? | 0.53 | 2.63 |
| *Richardoestesia* | TMP 1986.159.0062 | Sankey et al., 2002 | 3.50 | 1.50 | 7.50 | ? | 0.43 | 2.14 |
| *Richardoestesia* | TMP 1990.079.0031 | Sankey et al., 2002 | 2.80 | 1.30 | 6.80 | ? | 0.46 | 2.43 |
| *Richardoestesia* | TMP 1995.177.0049a | Sankey et al., 2002 | 3.20 | 1.20 | 8.50 | ? | 0.38 | 2.66 |
| *Richardoestesia* | TMP 1995.180.0005a | Sankey et al., 2002 | 1.90 | 0.90 | 5.20 | ? | 0.47 | 2.74 |
| *Richardoestesia* | TMP 1995.180.0005b | Sankey et al., 2002 | 1.70 | 0.90 | 4.20 | ? | 0.53 | 2.47 |
| *Richardoestesia* | TMP 1996.048.0011 | Sankey et al., 2002 | 2.50 | 1.30 | 5.00 | ? | 0.52 | 2.00 |
| *Richardoestesia* | TMP 1996.062.0030a | Sankey et al., 2002 | 3.60 | 1.70 | 12.50 | ? | 0.47 | 3.47 |
| *Richardoestesia* | TMP 1984.001.0012 | Sankey et al., 2002 | 4.50 | 2.10 | 11.00 | ? | 0.47 | 2.44 |
| *Richardoestesia* | TMP 1986.033.0054 | Sankey et al., 2002 | 2.10 | 1.10 | 4.50 | ? | 0.52 | 2.14 |
| *Richardoestesia* | TMP 1986.045.0046 | Sankey et al., 2002 | 2.30 | 1.20 | 5.50 | ? | 0.52 | 2.39 |
| *Richardoestesia* | TMP 1988.036.0199 | Sankey et al., 2002 | 3.70 | 1.70 | 9.50 | ? | 0.46 | 2.57 |
| *Richardoestesia* | TMP 1995.181.0010 | Sankey et al., 2002 | 3.00 | 1.60 | 7.20 | ? | 0.53 | 2.40 |
| *Richardoestesia* | TMP 1996.142.0019 | Sankey et al., 2002 | 3.10 | 1.50 | 9.70 | ? | 0.48 | 3.13 |
| *Richardoestesia* | TMP 1982.019.0366 | Farlow et al., 1991 | 3.10 | 1.80 | 6.00 | ? | 0.58 | 1.94 |
| *Richardoestesia* | LSUMG489:6237 | Sankey et al., 2005 | 2.00 | 0.90 | 2.50 | ? | 0.45 | 1.25 |
| *Richardoestesia* | LSUMG489:6235 | Sankey et al., 2005 | 1.70 | 0.70 | 3.00 | ? | 0.41 | 1.76 |
| *Richardoestesia* | LSUMG489:6050 | Sankey et al., 2005 | 1.70 | 1.00 | 3.50 | ? | 0.59 | 2.06 |
| *Richardoestesia* | LSUMG741:5933 | Sankey et al., 2005 | 1.80 | 0.90 | 2.30 | ? | 0.50 | 1.28 |
| *Richardoestesia* | LSUMG113:5939 | Sankey et al., 2005 | 2.00 | 1.00 | 4.00 | ? | 0.50 | 2.00 |

All measurements are in millimeters. For the teeth studied here: an asterisk denotes the latest previous published identification, and following is a our proposed identification when it differs.

Farlow JO, Brinkman DL, Abler WL, Currie PJ. Size, shape, and serration density of theropod dinosaur lateral teeth. Modern Geol. 1991;16: 161-198.

Hendrickx C, Mateus O, Araújo R. The dentition of megalosaurid theropods. Acta Palaeontol Polon. 2015;60: 627-642.

Longrich NR. Small theropod teeth from the Lance Formation of Wyoming, USA. In: Sankey JT, Baszio S, editors. Vertebrate Microfossil Assemblages: Their Role in Paleoecology and Paleobiogeography. Bloomington: Indiana University Press; 2008. pp. 135-158.

Sankey JT, Brinkman DB, Guenther M, Currie PJ. Small theropod and bird teeth from late cretaceous (late Campanian) Judith River group, Alberta. J Paleontol. 2002;76: 751-763.

Sankey JT, Standhardt BR, Schiebout BA. Theropod teeth from the Upper Cretaceous (Campanian-Maastrichtian), Big Bend National Park, Texas. In: Carpenter K, editor. The Carnivorous Dinosaurs. Bloomington: Indiana University Press; 2005. pp. 127-152.
