# Supplementary figures and images for: Synchrotron imaging of dentition provides insights into the biology of Hesperornis and Ichthyornis, the “last” toothed birds
Source: BMC Evol Biol. 2016 Sep 23;16:178. doi: 10.1186/s12862-016-0753-6 (PMC5034473; doi:10.1186/s12862-016-0753-6)

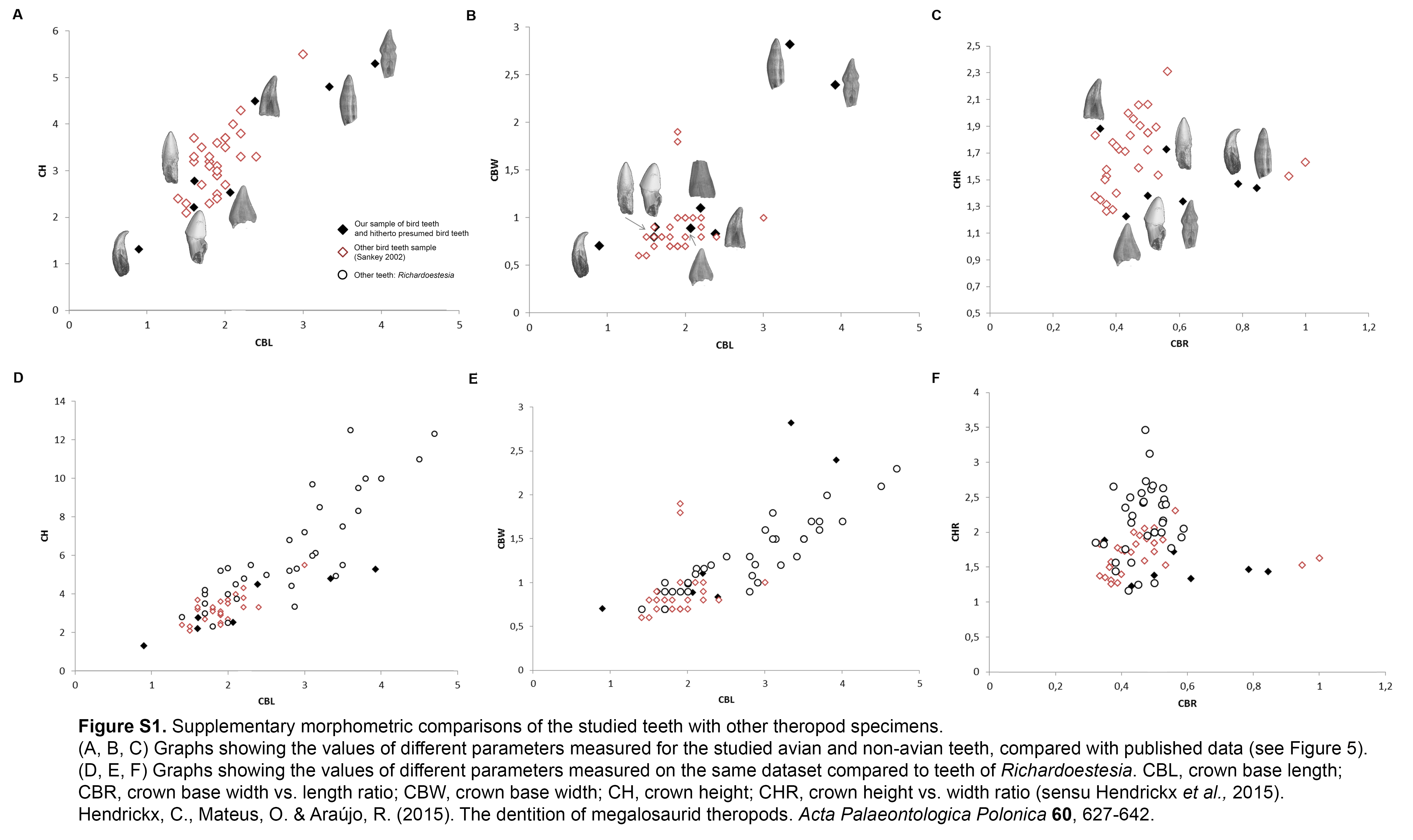

Supplement: Additional file 2: Fig. S1. — Supplementary morphometric comparisons of the studied teeth with other theropod specimens. (TIF 1082 kb) [file 12862_2016_753_MOESM2_ESM.tif]

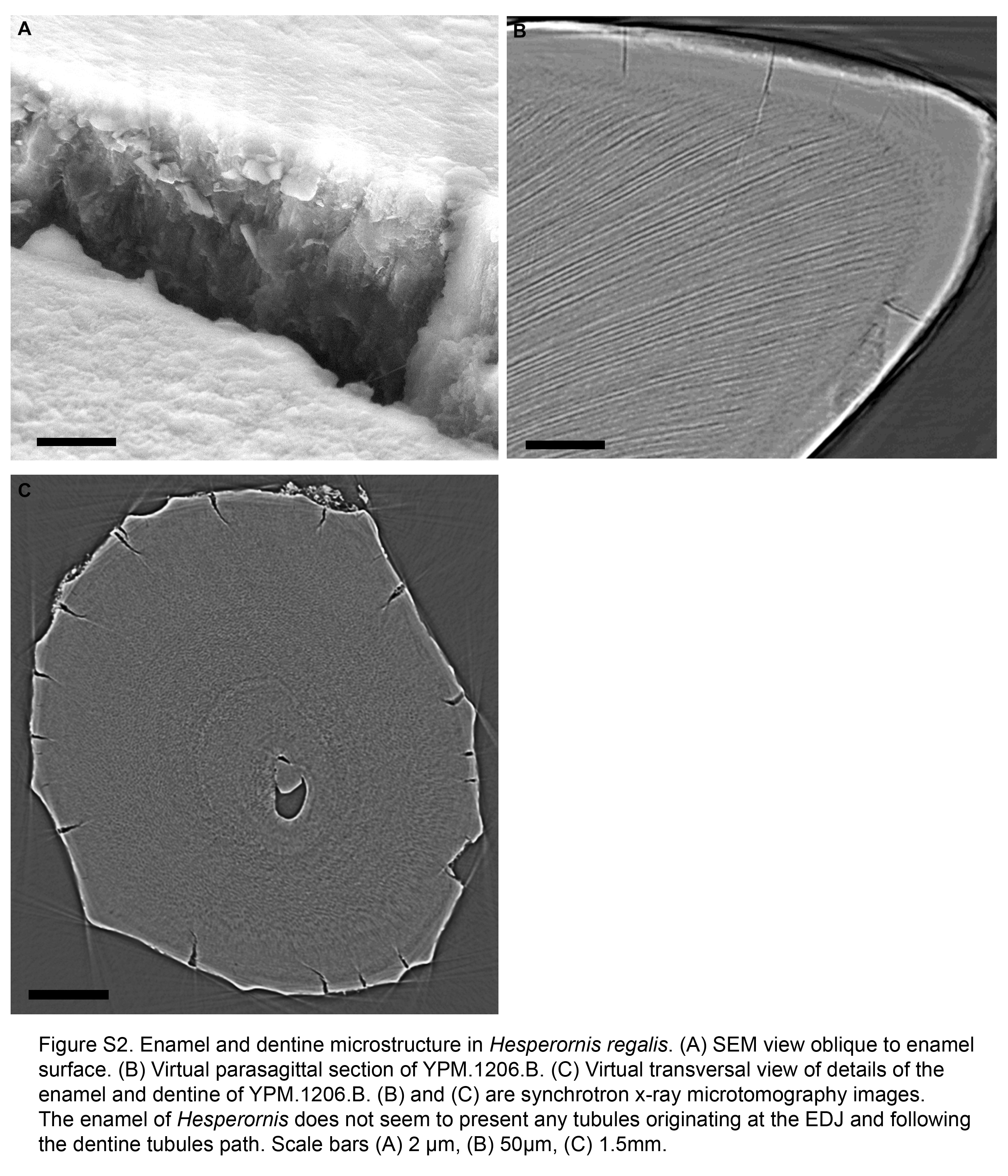

Supplement: Additional file 3: Fig. S2. — Enamel and dentine microstructure in Hesperornis regalis. (TIF 3290 kb) [file 12862_2016_753_MOESM3_ESM.tif]

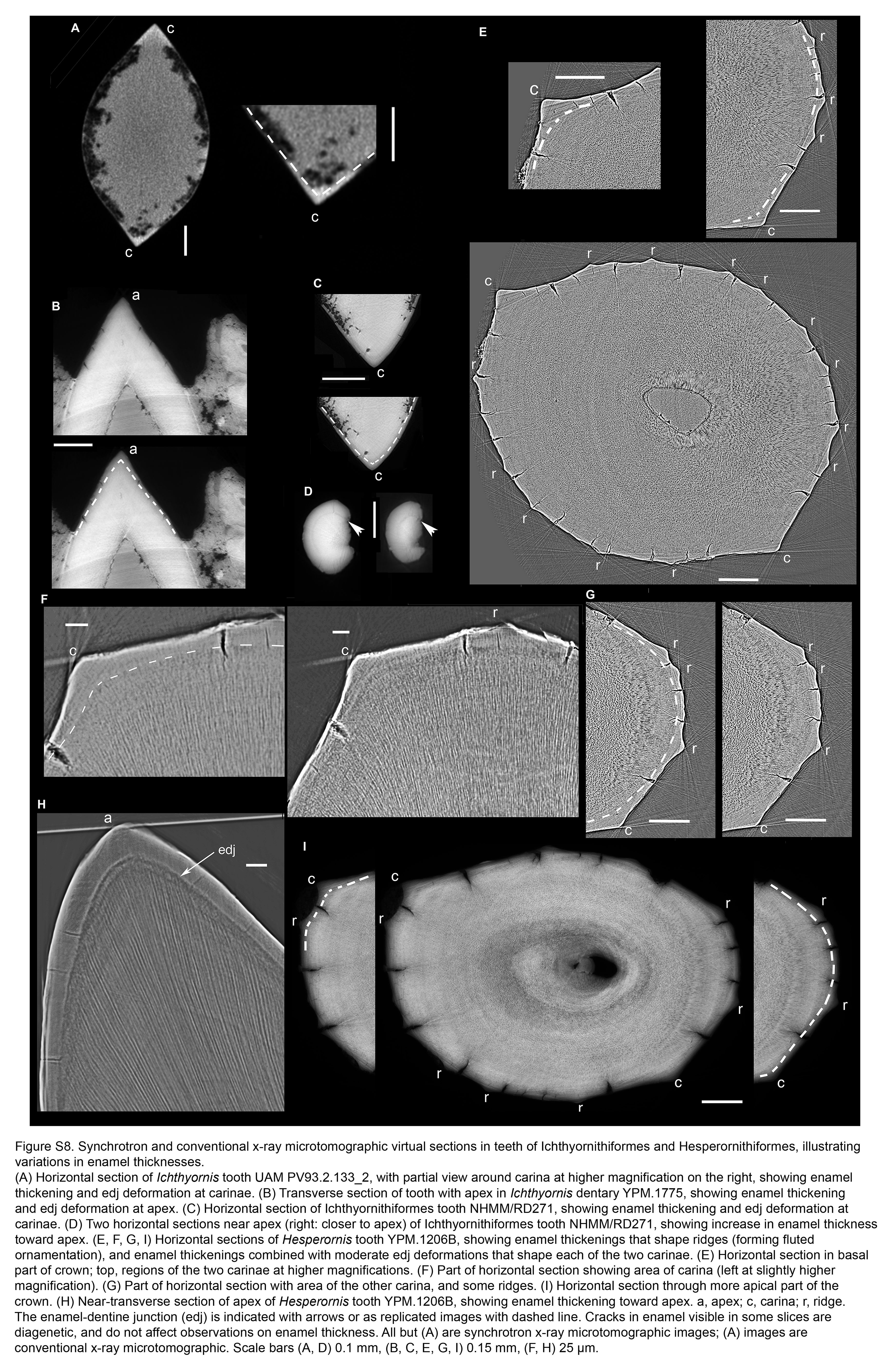

Supplement: Additional file 4: Fig. S8. — Synchrotron and conventional x-ray microtomographic virtual sections in teeth of Ichthyornithiformes and Hesperornithiformes, illustrating variations in enamel thicknesses. (TIF 9506 kb) [file 12862_2016_753_MOESM4_ESM.tif]

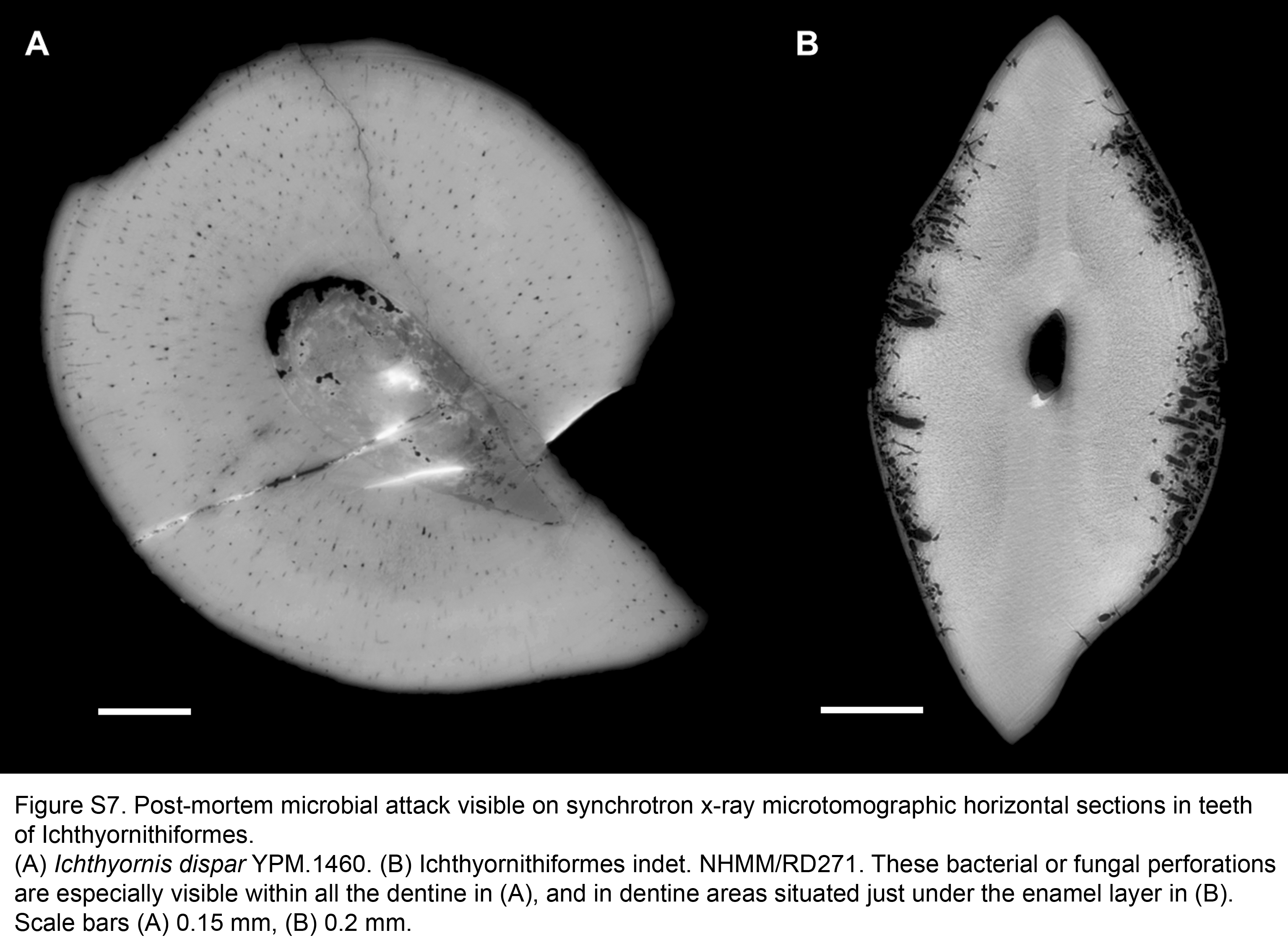

Supplement: Additional file 5: Fig. S7. — Post-mortem microbial attack visible on synchrotron x-ray microtomographic horizontal sections in teeth of Ichthyornithiformes. (TIF 1358 kb) [file 12862_2016_753_MOESM5_ESM.tif]

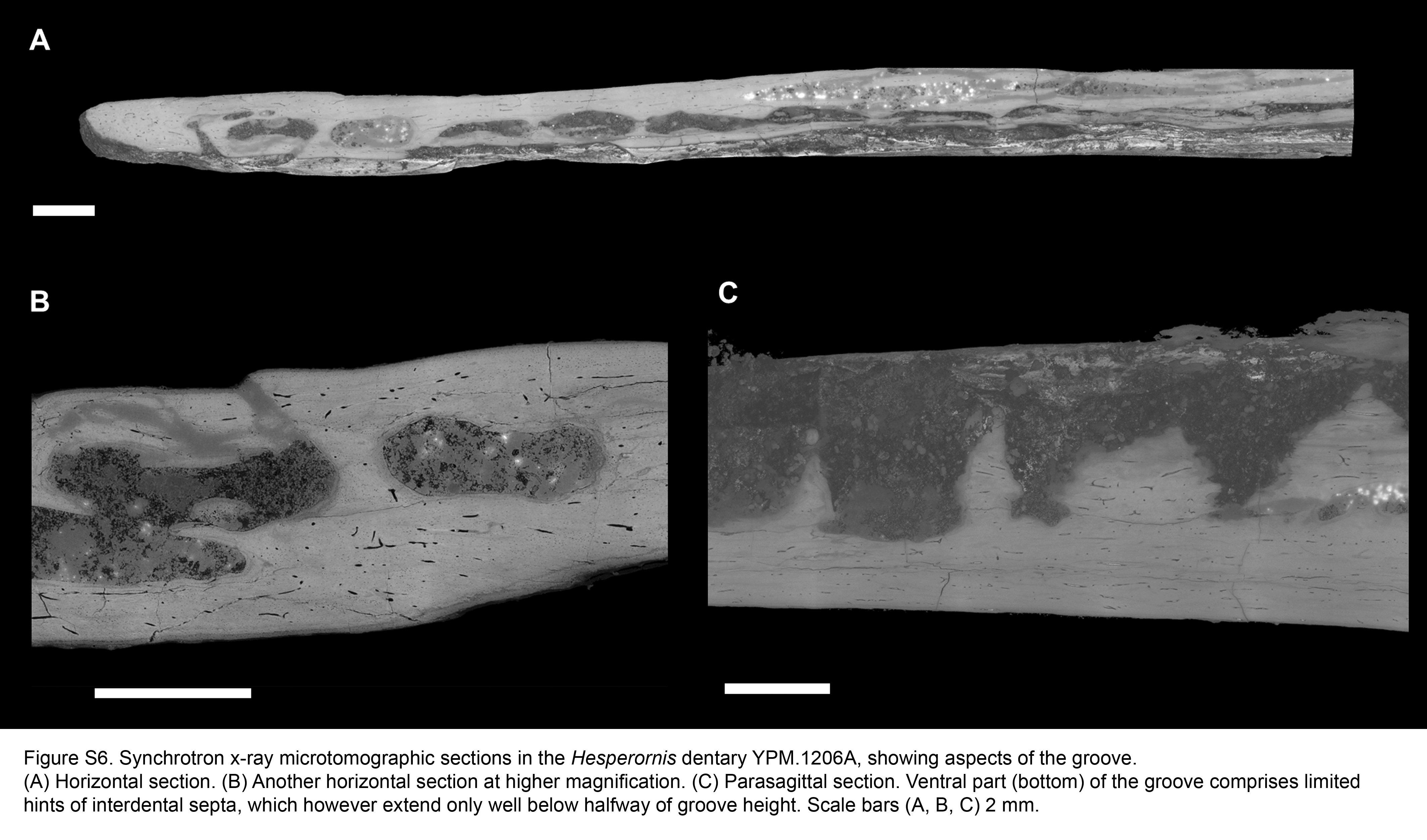

Supplement: Additional file 6: Fig. S6. — Synchrotron x-ray microtomographic sections in the Hesperornis dentary YPM.1206A, showing aspects of the groove. (TIF 2336 kb) [file 12862_2016_753_MOESM6_ESM.tif]

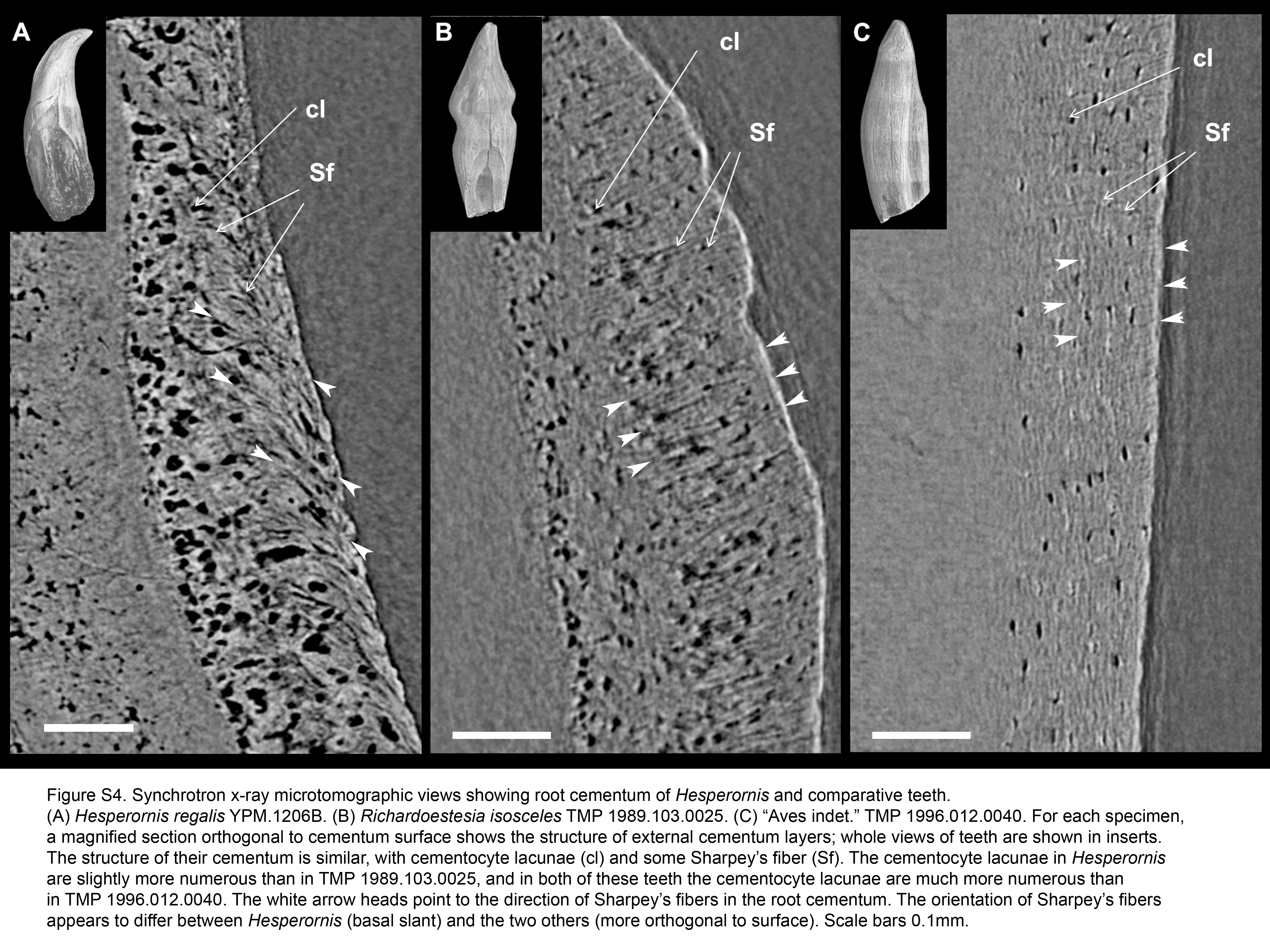

Supplement: Additional file 7: Fig. S4. — Synchrotron x-ray microtomographic views showing root cementum of Hesperornis and comparative teeth. (TIF 5049 kb) [file 12862_2016_753_MOESM7_ESM.tif]

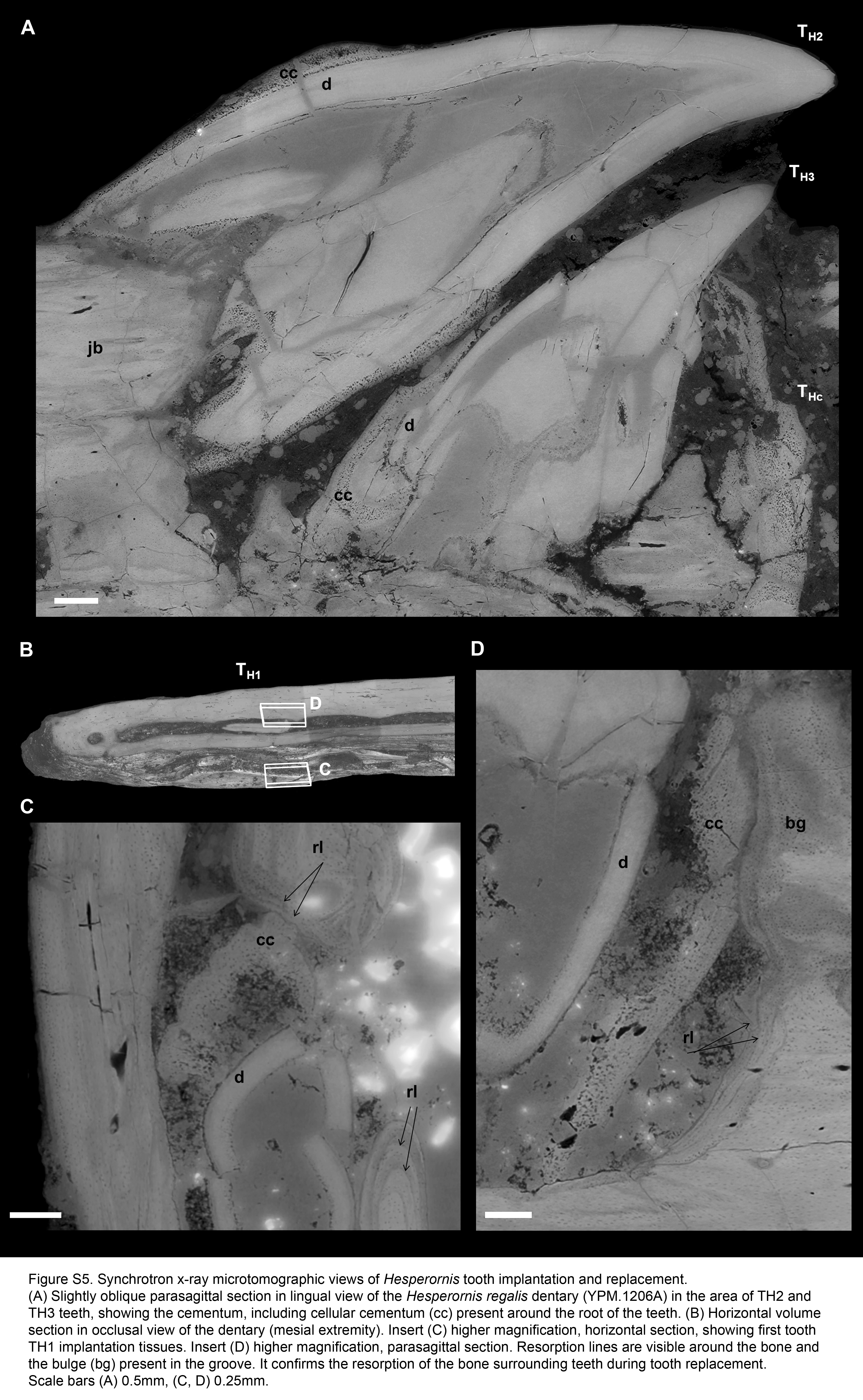

Supplement: Additional file 8: Fig. S5. — Synchrotron x-ray microtomographic views of Hesperornis tooth implantation and replacement. (TIF 7347 kb) [file 12862_2016_753_MOESM8_ESM.tif]

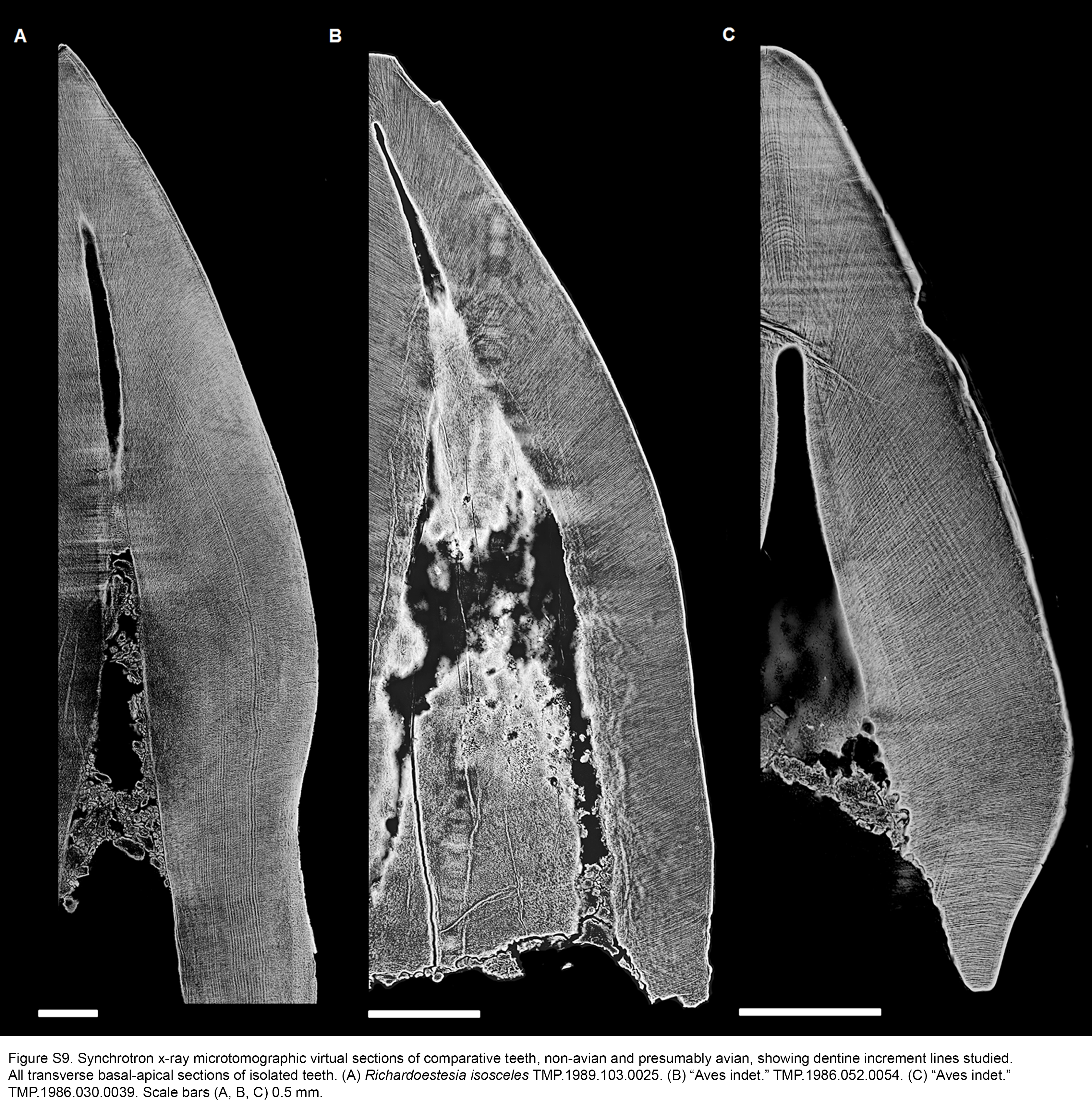

Supplement: Additional file 9: Fig. S9. — Synchrotron x-ray micromographic virtual sections of comparative teeth, non-avian and presumably avian, showing dentine increment lines studied. (TIF 8021 kb) [file 12862_2016_753_MOESM9_ESM.tif]

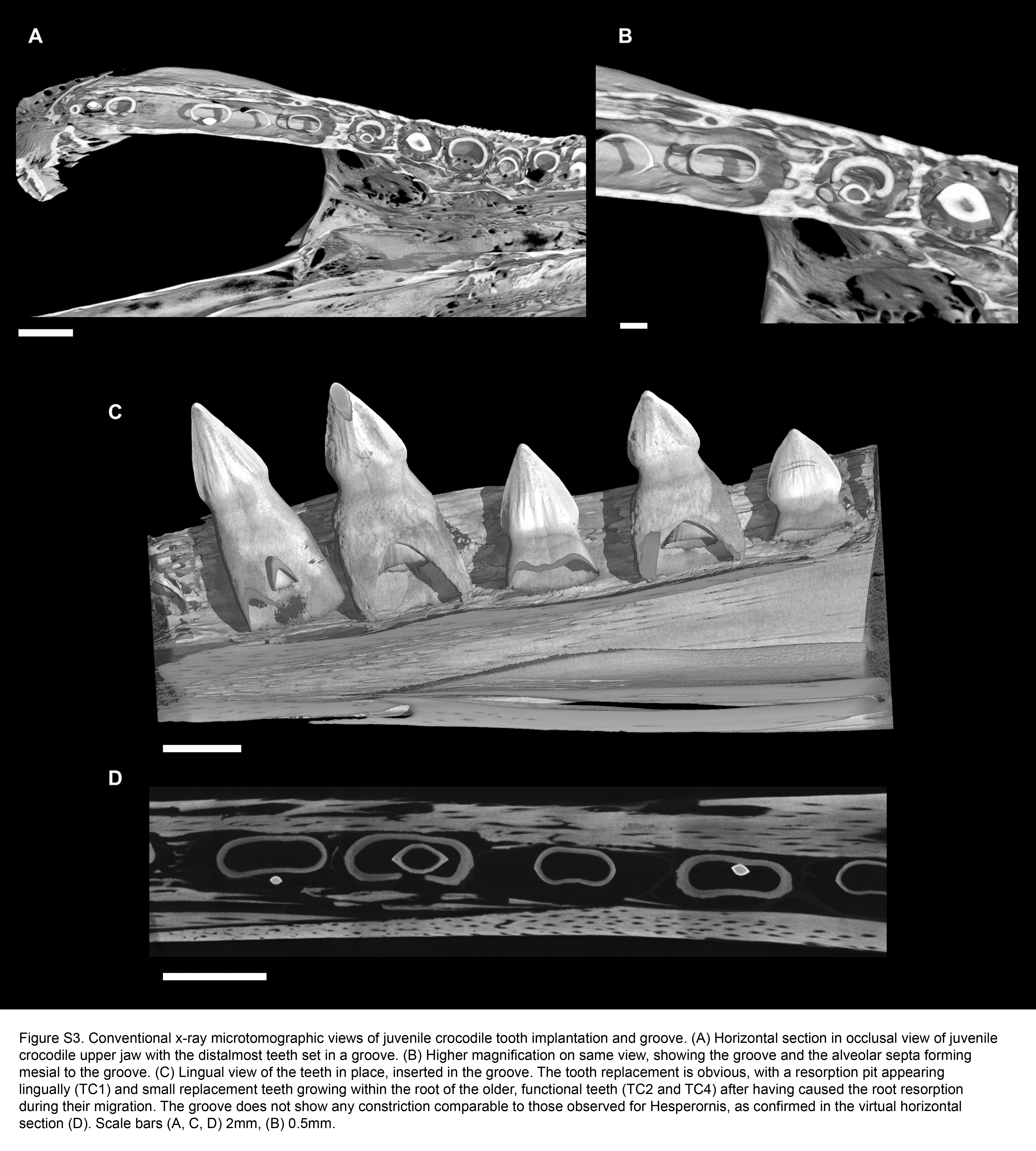

Supplement: Additional file 10: Fig. S3. — Conventional x-ray microtomographic views of juvenile crocodile tooth implantion and groove. (TIF 4109 kb) [file 12862_2016_753_MOESM10_ESM.tif]
